# On some new species of Stenheliinae Brady, 1880 (Copepoda, Harpacticoida, Miraciidae) from north-western Mexico, with the proposal of *Lonchoeidestenhelia* gen. nov.

**DOI:** 10.3897/zookeys.987.52906

**Published:** 2020-11-06

**Authors:** Samuel Gómez

**Affiliations:** 1 Instituto de Ciencias del Mar y Limnología, Unidad Académica Mazatlán, Universidad Nacional Autónoma de México, Joel Montes Camarena s/n, Fracc. Playa Sur, Mazatlán, 82040, Sinaloa, Mexico Universidad Nacional Autónoma de México Mazatlán Mexico

**Keywords:** Diversity, new genus, new species, pollution, taxonomy, *
Willenstenhelia
*

## Abstract

Quarterly sampling campaigns during 2019 to study the diversity of meiofauna in a polluted estuary in northwestern Mexico revealed the subfamily Stenheliinae Brady, 1880 as one of the most important contributors to the diversity of benthic harpacticoids. Two new stenheliin species are described here. One of them was assigned to the, so far, monotypic genus *Lonchoeidestenhelia***gen. nov.** defined by the autapomorphic modified proximal outer spinules on the sigmoid process of the male P2ENP2. The other species was assigned to *Willenstenhelia* Karanovic and Kim, 2014. *Lonchoeidestenhelia***gen. nov.** shares the armature formula of the P1 EXP2 with *Stenhelia*, *Anisostenhelia*, and *Beatricella*, but seems to bear a sister-group relationship with the former two genera by the loss of one inner seta on the P2–P3 EXP3, the presence of two outer spine-like elements on the male P5EXP, and the displacement of the outer spine and medial and inner distal setae of P2ENP3, to an apical and subapical inner position, respectively, but is more closely related to *Anisostenhelia* by the overall shape of the male P2ENP2. *Willenstenhelia reducta***sp. nov.** is attributed to a group of species composed of *Wi. minuta*, *Wi. urania*, and *Wi. terpsichore* characterized by the strongly reduced inner seta of the female P5 baseoendopod, but differs in the discrete female P5 baseoendopods and in the presence of one outer seta only on that segment. *Willenstenhelia reducta***sp. nov.** is defined here by the autapomorphic loss of the outermost seta of the female P5 baseoendopod.

## Introduction

A series of quarterly sampling campaigns were carried out during 2019 in the frame of a short-term project financed by the Universidad Nacional Autónoma de México, aiming at assessing the present effects of organic pollution on the distribution and diversity of meiofauna in an estuary in northwestern Mexico. Preliminary analyses revealed the subfamily Stenheliinae Brady, 1880 (Miraciidae Dana, 1846) to be one of the most important contributors to the overall diversity and density of benthic harpacticoids. In addition to the new species presented here, *Pseudostenhelia wellsi* Coull and Fleeger, 1977 was also found, although its overall contribution to the diversity and density of harpacticoid copepods in the study area was far less important. Here I present the description of two new stenheliin species. One of them was assigned to a new genus, *Lonchoeidestenhelia* gen. nov. as *L. prote* sp. nov., defined here by the autapomorphic modified proximal outer spinules on the sigmoid process of the male P2ENP2. Some comments on the relationships amongst *Stenhelia* Boeck, 1865, *Anisostenhelia* Mu and Huys, 2002, *Beatricella* Scott, 1905, and *Lonchoeidestenhelia* gen. nov. are given. The other species was assigned to *Willenstenhelia* Karanovic and Kim, 2014 as *Wi. reducta* sp. nov. Some comments on the relationships amongst the species of *Willenstenhelia* are given.

## Materials and methods

**Figure 1.**
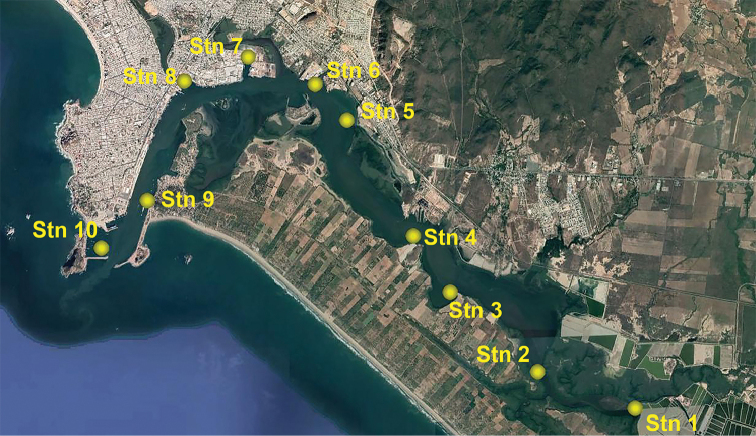
Sampling locations in Urías estuary, Mazatlán, Sinaloa State, Mexico. Coordinates as follows: stn 1 (23.15194°N, 106.3128°W); stn 2 (23.1587°N, 106.3326°W); stn 3 (23.1735°N, 106.3504°W); stn 4 (23.1840°N, 106.3579°W); stn 5 (23.2056°N, 106.3715°W); stn 6 (23.2123°N, 106.3780°W); stn 7 (23.2174°N, 106.3917°W); stn 8 (23.2128°N, 106.4047°W); stn 9 (23.1904°N, 106.4121°W); stn 10 (23.1815°N, 106.4214°W). Map data 2020 Google.

Sediment samples were taken from a series of sampling stations along Urías system (Fig. 1), a polluted estuary in southern Sinaloa State (north-western Mexico), using an Eckman grab of 25×25 cm (sampling surface, 625 cm^2^). Triplicate sediment cores were taken at each station using acrylic corers of 5.6 cm ID (24.6 cm^2^) and 20 cm in length, from which the upper 3 cm layer was retrieved. Each sample was fixed in pure ethanol and sieved through 500 µm and 38 µm sieves to separate macro- and meiofauna. Meiofauna was extracted through centrifugation with Ludox® HS-40 following Burgess (2001) and Rohal et al. (2016) and preserved in pure ethanol. Meiofauna was sorted at a magnification of 40× using an Olympus SZX12 stereomicroscope equipped with DF PLAPO 1× objective and WHS10× eyepieces, and harpacticoid copepods were stored separately in 1-ml vials containing pure ethanol.

Illustrations and figures were made from whole individuals and their dissected parts using a Leica DMLB microscope equipped with L PLAN 10× eyepieces, N PLAN 100× oil immersion objective, and drawing tube. The dissected parts were mounted on separate slides using lactophenol as mounting medium.

Huys and Boxshall (1991) was followed for general terminology. Abbreviations used in the text:

**acro** acrothek;

**ae** aesthetasc;

**BENP** baseoendopod;

**ENP** endopod;

**EXP** exopod;

**EXP/ENP1**(**2**,**3**) exopodal/endopodal 1^st^(2^nd^, 3^rd^) segment;

**P1**–**P6** first to sixth legs.

### Repositories

The type material was deposited in the Copepoda collection of the Instituto de Ciencias del Mar y Limnología, Unidad Académica Mazatlán (**ICML–EMUCOP**).

## Taxonomy

### Order Harpacticoida Sars, 1903

**Family Miraciidae Dana, 1846**

**Subfamily Stenheliinae Brady, 1880**

#### Genus Lonchoeidestenhelia gen. nov.

Taxon classificationAnimaliaHarpacticoidaMiraciidae

F287F928-9279-5D9F-9AAE-4D0FDBDC645F

http://zoobank.org/67AB6B9E-4DDA-45FB-A9E8-D68A716B750F

##### Type and only species.

*Lonchoeidestenhelia prote* sp. nov., by monotypy.

##### Diagnosis

(based on its only species, *L. prote* sp. nov.). Stenheliinae. ***Female***: Anal somite twice as long as broad; anal operculum rounded, with minute spinules along its posterior margin, with one sensillum on each side. Caudal rami cylindrical, twice as long as broad, and slightly longer than anal somite; with seven setae, of which seta I spine-like, setae IV and V with fracture plane, rat-tail-like. Rostrum trapezoidal, not fused to cephalothorax, bifid, with medial pore and two subdistal sensilla. Antennule seven-segmented; all setae smooth except for one and two pinnate setae on first and second segments; second and third segments each with one seta with fracture plane; sixth and seventh segments each with two articulated setae; aesthetasc on segments 4 and 7. Antenna with allobasis; exopod three-segmented, armature formula 1,1,[1 lateral + 3 apical, two of which fused basally]. Mandible with elongate basis tapering distally; exopod arising from short pedestal, one-segmented, ca. 4× as long as wide, tapering distally, with three lateral and three apical slender setae; endopod recurved, twisted over exopod, with three lateral setae, and five distal elements (one short seta and long pinnate element fused basally and to endopod, one slender pinnate and one strong bare element, and one long element fused to endopod and with hyaline flange in middle part). Maxillule without modified elements on arthrite; basis with two endites, proximal endite with four, distal endite with three slender setae; exopod and endopod fused basally, separated from basis, one-segmented, endopod with four setae, exopod with two setae. Maxilliped subchelate; syncoxa with one bare and two spinulose strong elements; basis with two slender distal setae unequal in length; endopod one-segmented, slender, with one claw and one accompanying seta. Armature seta of P1–P4 as follows:

**Table:** 

	P1	P2	P3	P4
**EXP**	0,0,022	1,1,123	1,1,223	1,1,323
**ENP**	1,1,111	1,2,121	1,1,221	1,1,221

P5 baseoendopod transversely elongate, with five setae, all setae normal; exopod with six setae. ***Male***: Habitus, anal somite, and caudal rami as in female. Sexual dimorphism expressed in A1, P2ENP, P5, and P6. Antennule 10-segmented, haplocer; all segments smooth except for first and seventh segment with spinules; all setae smooth except for one and two pinnate setae on first and second segments, and one and two modified spine-like elements on seventh and eighth segments; aesthetasc on third, fifth, and tenth segments. P2ENP2 transformed, proximal half globular, distal half triangular, without hyaline flange, with two inner (one proximal and one medial) small setae, one inner subdistal strong pinnate element, and one apical sigmoid pinnate process with proximal row of modified (lanceolate) outer spinules. Baseoendopods of both P5 fused medially, each endopodal lobe with two setae, of which the inner well-developed, the outer small; exopod small, discrete, with four elements, of which two outermost spine-like. P6 asymmetrical, only one leg functional, each leg with three normal setae.

##### Etymology.

The genus name from the Greek *λογχοειδής*, lonchoeidḗs̱, lanceolate, makes reference to the shape of the proximal spinules on the distal outer process of the male P2ENP2. Gender feminine.

#### Lonchoeidestenhelia prote sp. nov.

Taxon classificationAnimaliaHarpacticoidaMiraciidae

C419887A-9107-5049-8DEA-3E0BE562CCBB

http://zoobank.org/D1A4C714-C914-40D5-B141-4CA9828E2BEE

Figs 2 , 3 , 4 , 5 , 6 , 7 , 8 , 9 , 10 , 11

##### Specimens examined.

One female holotype (ICML–EMUCOP-180119-01), one male allotype (ICML–EMUCOP-180119-02), and 14 paratypes (10 females and four males) (ICML–EMUCOP-180119-03) preserved in alcohol, and two female (ICML–EMUCOP-180119-05, ICML–EMUCOP-180119-06) and one male (ICML–EMUCOP-180119-07) paratypes dissected and mounted onto 11, six and seven slides, respectively, all from the type locality; six paratypes (two females and four males) (ICML–EMUCOP-180119-04) from stn 1, preserved in alcohol; one female paratype partially dissected (ICML–EMUCOP-180119-08) (P1-P4 dissected and mounted onto one slide, the rest left intact and preserved in alcohol), and nine paratypes (eight females and one male) (ICML–EMUCOP-180119-09) from stn 10, preserved in alcohol; 18 Jan. 2019. S. Gómez leg.

##### Additional material examined.

One intersexual individual partially dissected (ICML–EMUCOP-180119-10) (P1-P4 dissected and mounted onto one slide, the rest left intact and preserved in alcohol) from stn 4; 18 Jan. 2019. S. Gómez leg.

##### Differential diagnosis.

Stenheliinae. Anal operculum present, with minute spinules along posterior margin. P1 EXP2 without inner armature. P2–P4 EXP1 with inner seta. Armature formula of P2 EXP3 and P3 EXP3, 123 and 223, respectively. Female P2ENP3 with outer apical spinous process, with displacement of medial and inner apical setae to subapical inner margin, the latter setae normal, not swollen basally. Female P5 baseoendopod without modified setae. Male P2ENP2 without hyaline flange; outer apical sigmoid, bipinnate, flagellate process with incomplete suture indicating original articulation with the supporting segment, with longitudinal row of modified lanceolate spinules proximally. Outer spine of the male P4ENP3 normal, not sexually dimorphic. Male P5EXP and baseoendopod not fused; exopod with two outermost elements modified into spines. Innermost seta of the male P6 normal.

##### Description.

**Figure 2.**
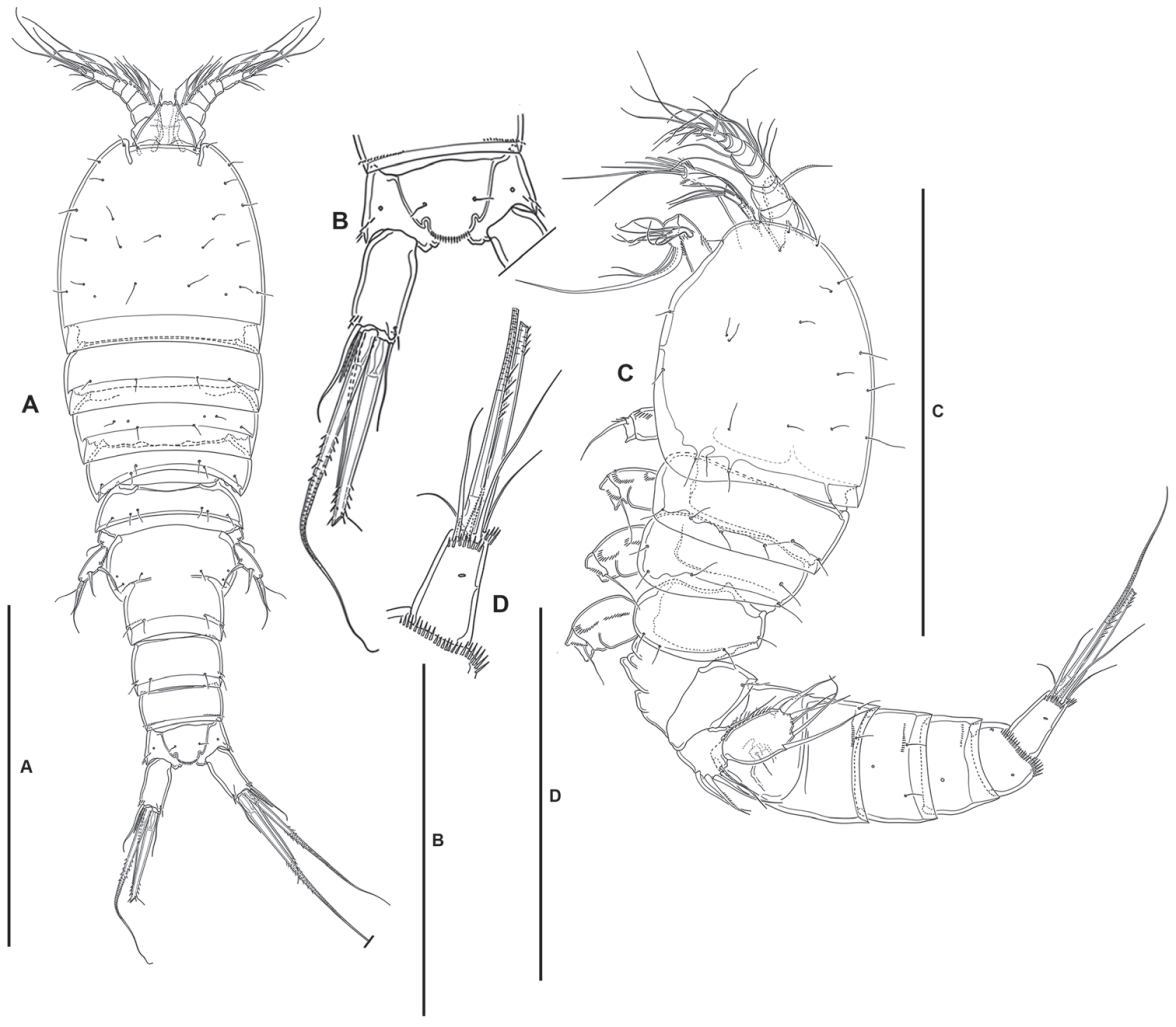
*Lonchoeidestenhelia prote* gen. et sp. nov., female **A** habitus, dorsal **B** anal somite and left caudal ramus, dorsal **C** habitus, lateral **D** left caudal ramus, lateral. Scale bars: 200 µm (**A, C**); 100 µm (**B, D**).

***Female*.** Total body length measured from tip of rostrum to posterior margin of caudal rami ranging from 415 µm to 563 µm (mean, 491 µm; n, 12; total body length of holotype, 563 µm); habitus pyriform, widest at posterior end of cephalothorax in dorsal view, tapering posteriad (Fig. 2A).

**Prosome** (Fig. 2A, B): Consisting of cephalothorax with fused first pedigerous somite, and second to fourth free pedigerous somites, the latter without expansions nor spinular ornamentation; posterior hyaline frill of cephalothorax and pedigerous somites plain, of fourth pedigerous somite visibly narrower; integument smooth, weakly sclerotized.

**Figure 3.**
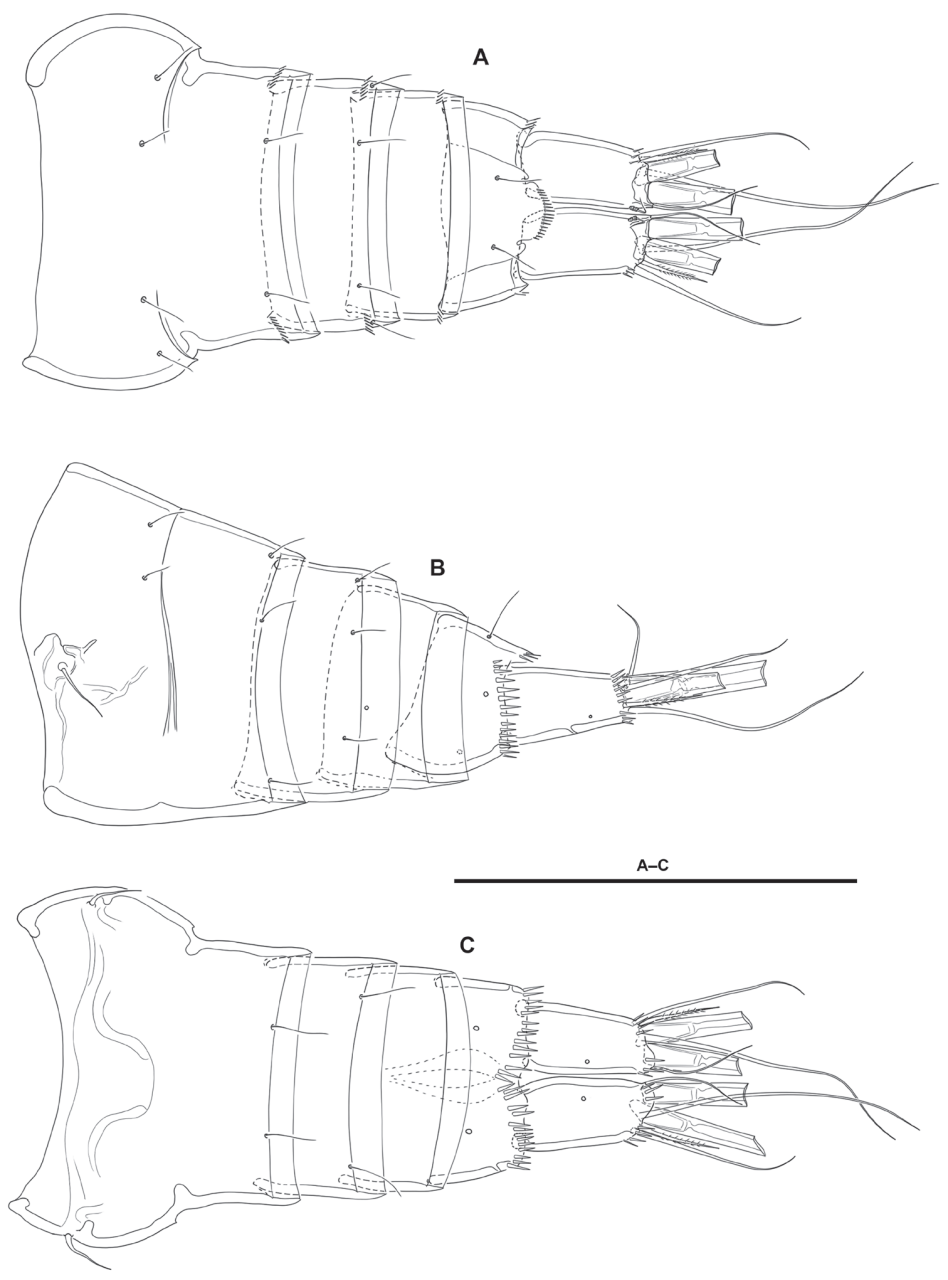
*Lonchoeidestenhelia prote* gen. et sp. nov., female **A** urosome, dorsal (P5–bearing somite omitted) **B** urosome, lateral (P5–bearing somite omitted) **C** urosome, ventral (P5–bearing somite omitted). Scale bars: 100 µm (**A–C**).

**Urosome** (Figs 2A, B, 3A–C): Consisting of fifth pedigerous somite (first urosomite), genital double-somite (genital (second urosomite) and third urosomites fused), two free urosomites, and anal somite; urosomites without expansions laterally nor dorsally; integument weakly sclerotized.

**First urosomite** (fifth pedigerous somite): Visibly narrower than preceding somites in dorsal view (Fig. 2A), without spinular ornamentation, with dorsolateral sensilla as shown (Fig. 2A, C).

**Genital double-somite**: Slightly wider than long, widest at proximal half; with dorsolateral internal rib marking original division between second (genital) and third urosomite (Figs 2A, C, 3A, B), completely fused dorsally (Figs 2A, 3A) and ventrally (Fig. 3C); proximal half (genital somite) without spinular ornamentation, with posterior sensilla as depicted; distal half (third urosomite) with short transverse row of small dorsolateral spinules (Figs 2A, 3A), with sensilla as shown.

**Fourth urosomite**: With spinular ornamentation (Fig. 3A–C) as in distal half of genital double-somite, with sensilla as shown.

**Fifth urosomite**: With spinular ornamentation (Fig. 3A–C) as in distal half of genital double-somite, without sensilla.

**Anal somite** (Figs 2A–C, 3A–C): Twice as long as broad, maximum breadth measured proximally; maximum length measured at the middle from anterior margin of somite to distal margin of anal operculum, with row of dorsolateral spinules close to joint with caudal rami, with one lateral pore on each side (Figs 2C, 3A, B); anal operculum rounded, with minute spinules along its posterior margin, with one sensillum on each side; ventrally cleft medially, with one pore on each side, with spinular row close to joint with caudal rami (Fig. 3C).

**Caudal rami** (Figs 2A–D, 3A–C): Typically divergent (Fig. 2A, B), but sometimes parallel (Fig. 3A, C), cylindrical, twice as long as broad, and slightly longer than anal somite; each ramus with one lateral (Fig. 2C, D) and one ventral pore (Fig. 3B, C); with spinules at base of setae I and II (Figs 2C, D, 3B), and ventrally close to insertion site of seta III (Fig. 3C); with seven elements as follows: seta I spine-like, ventral to seta II, the former visibly shorter, both arising subdistally on lateral margin; seta III ventral, subdistal, slightly longer than seta II; seta IV and V situated distally, with fracture plane, rat-tail-like; seta VI issuing at inner distal corner; dorsal seta VII triarticulate at base, situated subdistally close to inner margin.

**Figure 4.**
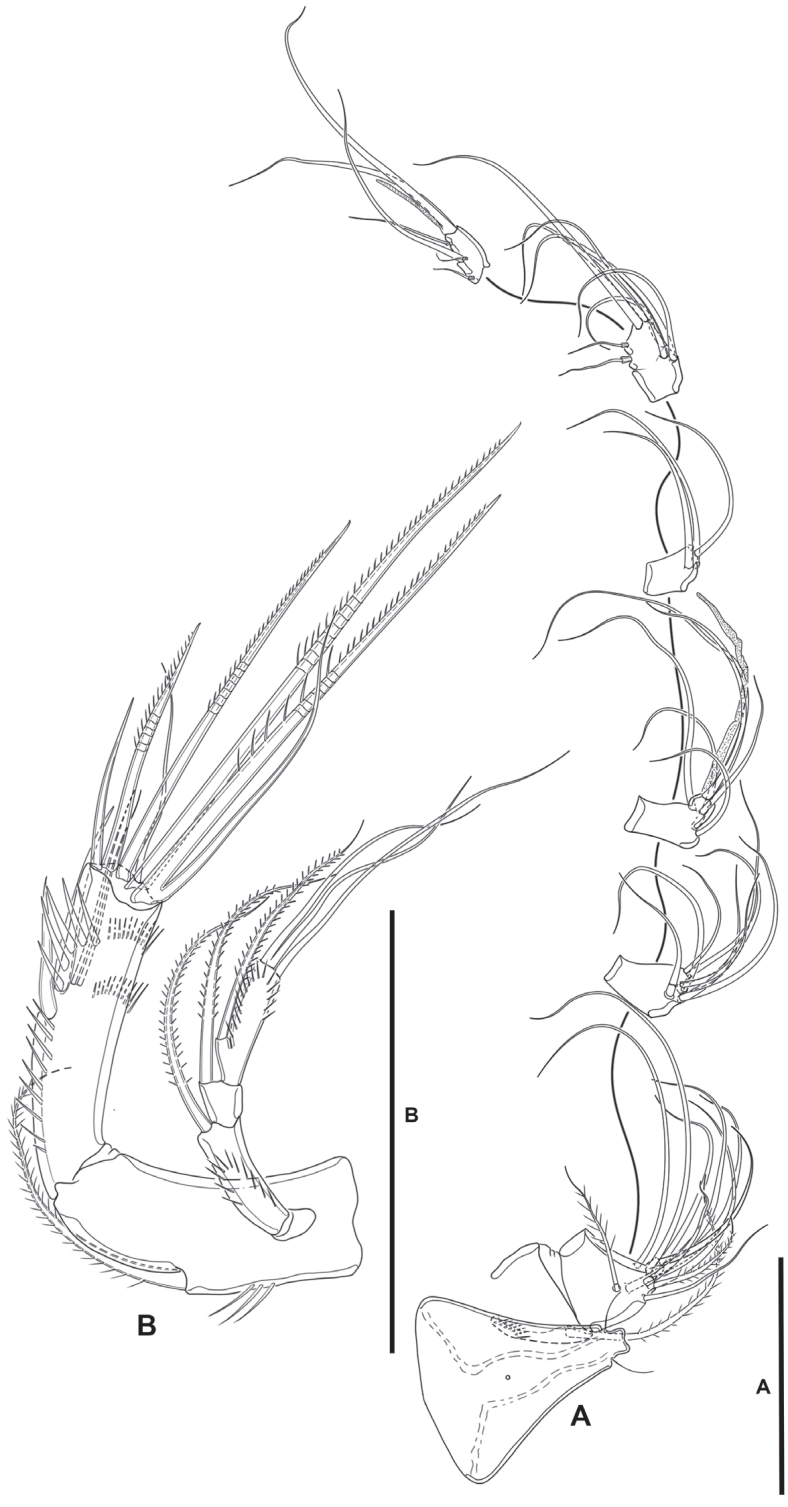
*Lonchoeidestenhelia prote* gen. et sp. nov., female **A** rostrum and antennule **B** antenna. Scale bars: 50 µm (**A, B**).

**Rostrum** (Figs 2A, 4A): Trapezoidal, not fused to cephalothorax, reaching middle of second antennular segment, bifid, with medial pore and two subdistal sensilla.

**Antennule** (Fig. 4A): Seven-segmented; all segments smooth, except for first segment with spinular row. All setae smooth except for one and two pinnate setae on first and second segments, respectively; second and third segments each with one seta with fracture plane; sixth and seventh segments each with two articulated setae. Armature formula: 1(1); 2(11); 3(8); 4(5+(1+ae)), 5(3); 6(8); 7(4+acro). Acrothek consisting of two setae and one slender aesthetasc fused at their bases.

**Antenna** (Fig. 4B): Allobasis as long as free endopodal segment, inner margin with long spinules on proximal third, with one abexopodal seta arising midway inner margin. Exopod three-segmented, issuing proximally; first and third segments longest, each 3× as long as wide, second segment shortest, ca. 1.5× as long as broad; first and second segments with one subdistal pinnate seta each, first segment with spinular row as shown, second segment unornamented; third segment with one lateral proximal pinnate element, and three distal setae, of which two fused basally, with spinular row as depicted. Free endopodal segment elongate, inner margin with spinular row proximally, subdistally with curved row of strong spinules, with medial and subdistal outer fringes; armature consisting of two lateral spines and two accompanying setae, one non-geniculate inner distal element, three distal geniculate spines (of which innermost shortest) and one slender seta, and one outer distal geniculate seta fused basally to slender element.

**Figure 5.**
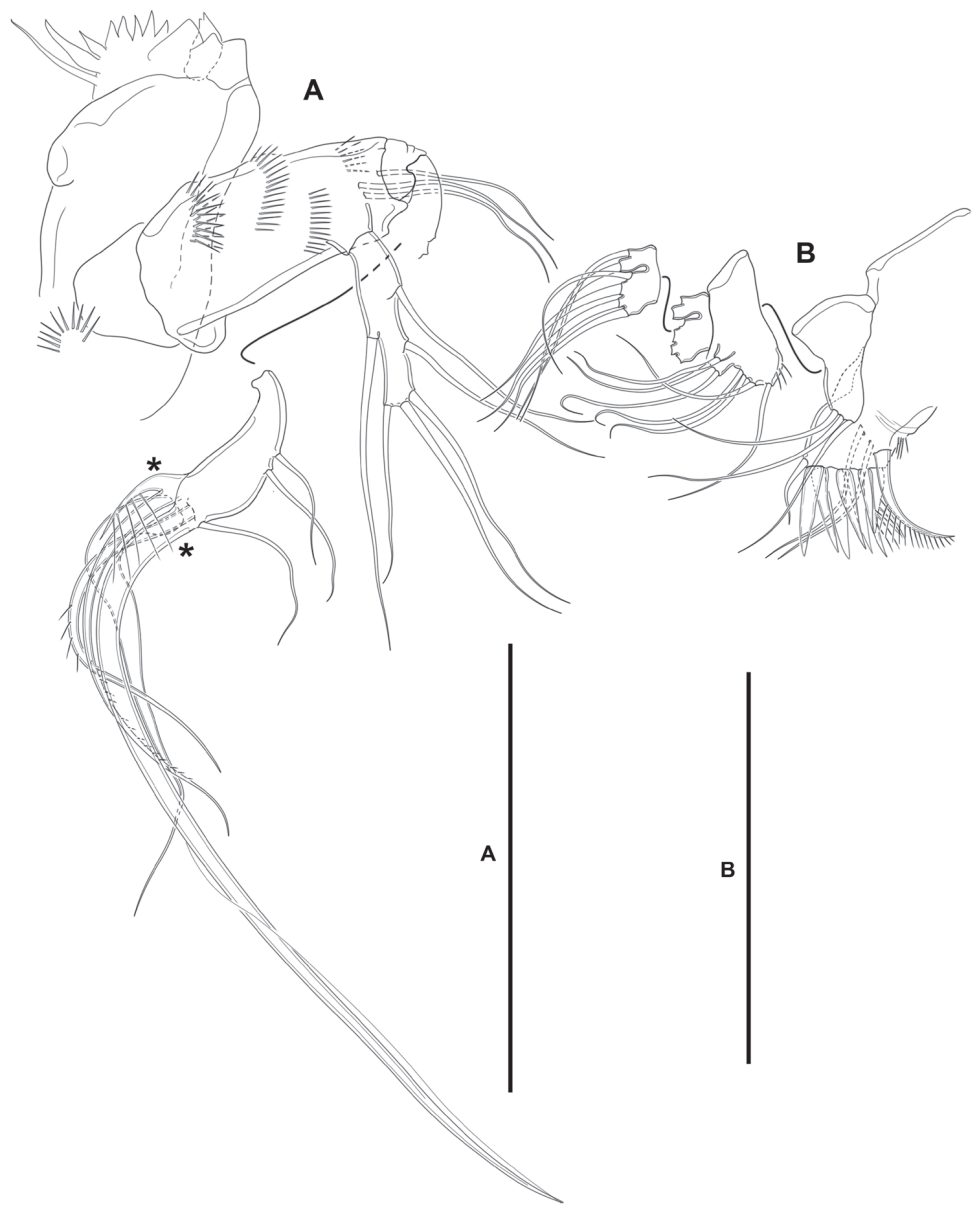
*Lonchoeidestenhelia prote* gen. et sp. nov., female **A** mandible (asterisks indicate two elements fused basally and to ramus, and single element fused to ramus) **B** maxillule. Scale bars: 50 µm (**A, B**).

**Mandible** (Fig. 5A): With relatively short coxa. Gnathobasis wide; with two strong bicuspidate teeth, several smaller bicuspidate teeth, some spinules, and one lanceolate element accompanied by seta. Basis elongate, tapering distally; with transverse spinular rows as shown; with three subdistal setae. Exopod arising from short pedestal, one-segmented, elongate, ca. 4× as long as wide, tapering distally; with three lateral and three apical slender setae, none of which fused basally. Endopod recurved, twisted over exopod; with three lateral setae, and five distal elements (one short seta and long pinnate element fused basally and to endopod, indicated with an asterisk in Fig. 5A, one slender pinnate and one strong bare element, and one long element fused to endopod and with hyaline flange in middle part, indicated with an asterisk in Fig. 5A).

**Maxillule** (Fig. 5B): Arthrite of praecoxa with two surface setae and seven bare distal elements (one of which a slender seta arising next to ventralmost spine), one spinulose dorsal spine, and one lateral spinulose recurved seta. Coxal endite with three setae. Basis with two endites; proximal endite with four, distal endite with three slender setae. Exopod and endopod fused basally, separated from basis, one-segmented; endopod larger than exopod, with four setae; exopod small, with two setae.

**Figure 6.**
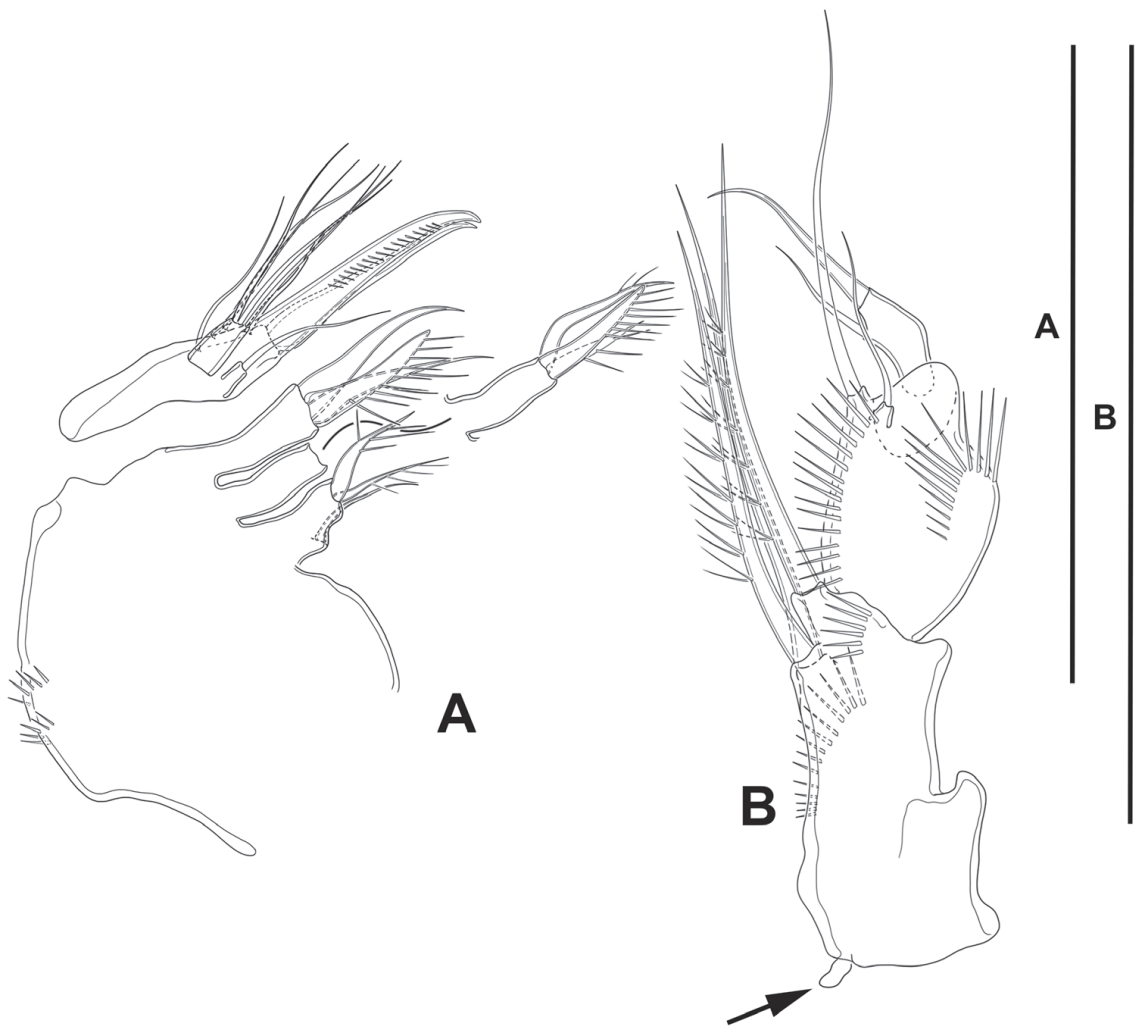
*Lonchoeidestenhelia prote* gen. et sp. nov., female **A** maxilla **B** maxilliped (arrow indicates minute intercoxal sclerite). Scale bars: 50 µm (**A, B**).

**Maxilla** (Fig. 6A): With large syncoxa with outer spinules as shown; with three endites; proximal endite smallest, with one proximal and two distal setae; middle and distal endites elongate, the latter slightly longer, with three elements each as figured. Basis drawn out into strong claw, additionally with strong spine and two slender setae, one of which arising from elongate setophore. Endopod one-segmented, 2× as long as wide, with six slender setae.

**Maxilliped** (Fig. 6B): Subchelate. Syncoxa rectangular, ca. 1.5× as long as wide, outer margin irregular and with medial protrusion; with spinules as shown; with one bare and two spinulose strong elements, of which the bare seta and one spinulose element subdistal and both at the same level, the other arising distally from long pedestal. Basis shorter than syncoxa, oval; with inner and outer spinules as depicted, and two slender distal setae, one of which visibly shorter. Endopod one-segmented, slender, with one claw-like element and one seta.

**Figure 7.**
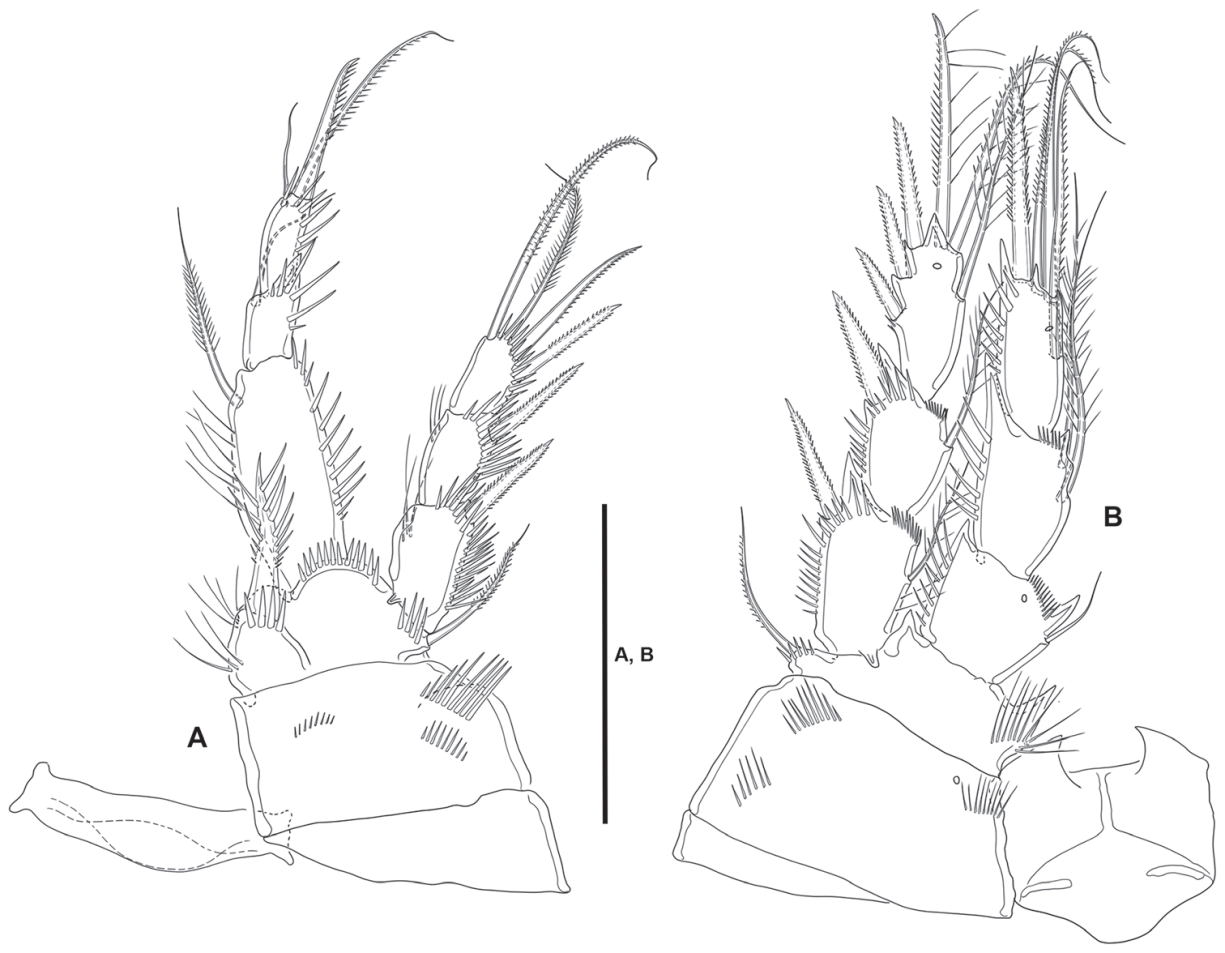
*Lonchoeidestenhelia prote* gen. et sp. nov., female **A**P1, anterior **B**P2, anterior. Scale bars: 50 µm (**A, B**).

**P1** (Fig. 7A): Intercoxal sclerite narrow and elongate, without surface ornamentation. Praecoxa large, triangular, unornamented. Coxa quadrate, with spinular rows as shown. Basis with inner robust and strongly spinulose spine, and outer slender pinnate element; with strong spinules at the bases of inner and outer elements and between rami, and with long slender inner spinules. Exopod three-segmented, reaching tip of first endopodal segment, situated at a lower level than ENP; first segment longest, third segment shortest; all segments without outer nor inner distal processes; with spinular ornamentation as shown; first and second segments without inner seta, third segment with four elements. Endopod three-segmented, situated distally on medial circular outgrowth of basis and at a higher level than EXP; ENP1:EXP length ratio 0.9, ENP1 ca. 1.4× as long as ENP2 and ENP3 combined; ENP1 and ENP3 without, ENP2 with outer acute distal process; segments with spinular ornamentation as figured; ENP1 with pinnate inner seta arising subdistally; ENP2 with one slender inner seta; ENP3 with three elements (one slender inner seta, one apical pinnate element, and one apical outer spine.

**P2** (Fig. 7B): Intercoxal sclerite not transversely elongate, trapezoidal, with strong pointed process on distal outer corners, without surface ornamentation. Praecoxa small, triangular, unornamented. Coxa massive, quadrate, with outer spinules proximally and subdistally, with subdistal spinules and one pore close to inner margin. Basis with outer setiform element and strong acute process between rami and on inner distal corner, the latter with slender spinules proximally. Exopod three-segmented, reaching slightly beyond ENP3; EXP1 and EXP2 with inner distal frill, with outer acute distal process, with spinular ornamentation as shown, and with one inner seta; EXP3 with processes as shown, with small outer spinules at base of proximal outer spine, with subdistal pore, with one inner and two apical setae, and three outer spines. Endopod three-segmented; segments with spinules as shown; ENP1 shortest, with subdistal inner pore, with inner and outer acute processes, the former slightly larger, with one slender short inner seta; ENP2 and ENP3 subequal in length, the former with outer acute and small inner process, with two inner setae subequal in length; ENP3 with distal processes as shown, with subdistal inner pore, with one inner seta, two inner apical elements, and one outer distal spine.

**Figure 8.**
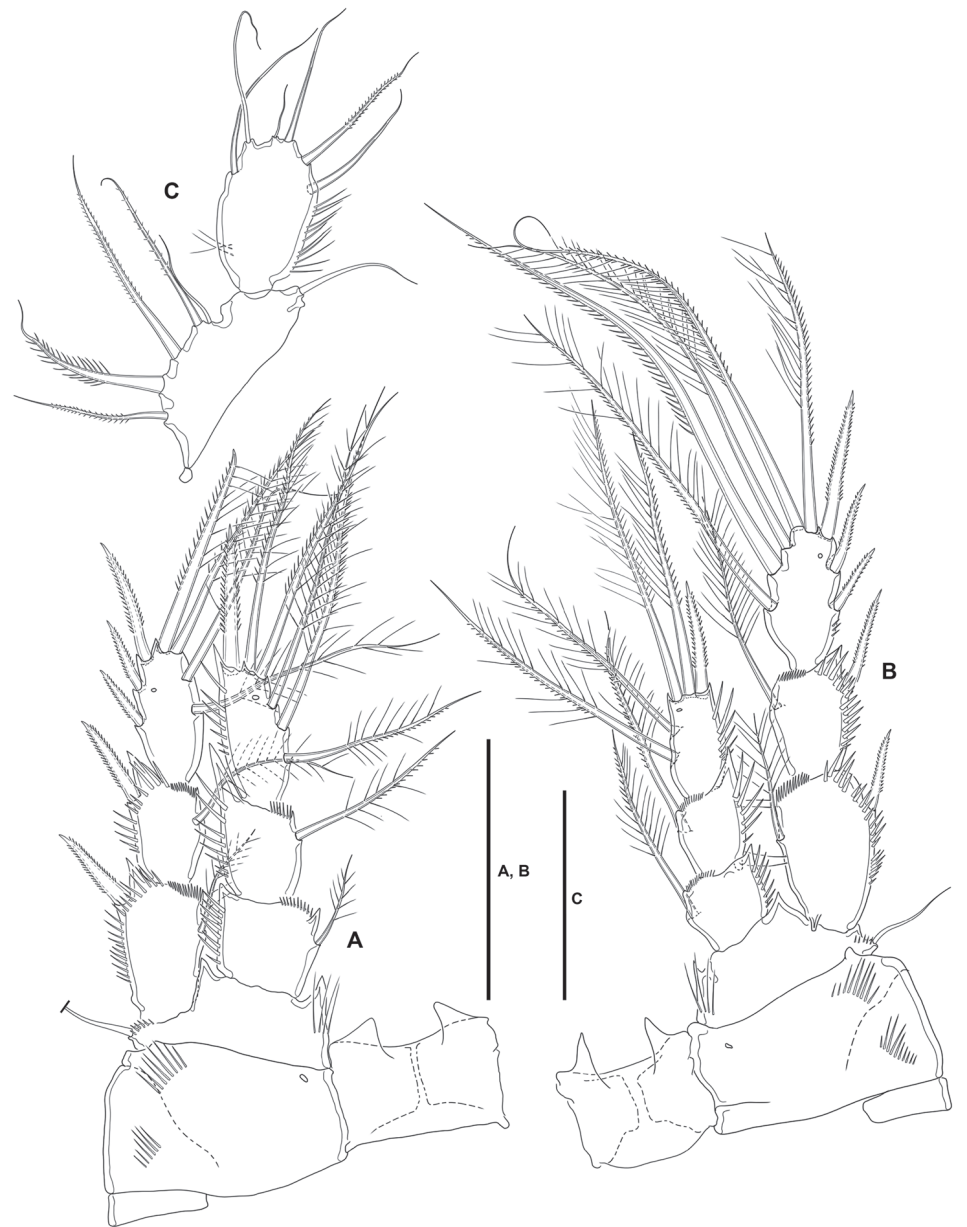
*Lonchoeidestenhelia prote* gen. et sp. nov., female **A**P3, anterior **B**P4, anterior **C**P5, anterior. Scale bars: 50 µm (**A–C**).

**P3** (Fig. 8A): Intercoxal sclerite not transversely elongate; trapezoidal; wider than in P2; with strong pointed process on distal outer corners; without surface ornamentation. Praecoxa triangular, small. Coxa as in P2 but without inner spinules. Basis as in P2, but with somewhat more slender outer seta. Exopod three-segmented, slightly longer than ENP; segments with spinules as shown; EXP1 and EXP2 with outer acute distal process, without pores, with inner distal frill, and with one inner seta each; EXP3 with outer subdistal pore, with two inner setae, two apical elements, and three outer spines. Endopod three-segmented; spinular ornamentation of segments as depicted; ENP1 shortest, ENP3 longest; ENP1 with small outer and inner distal processes, with inner seta; ENP2 with well-developed outer and small inner distal process, with inner pinnate seta; ENP3 with distal processes as shown, with subdistal outer pore, with two inner and two apical setae, and one outer apical spine.

**P4** (Fig. 8B): Intercoxal sclerite not transversely elongate; trapezoidal; smaller than in P3; with strong pointed process on distal outer corners; without surface ornamentation. Praecoxa, coxa and basis as in P3 except for comparatively smaller inner distal process of basis. Exopod three-segmented, longer than ENP; spinular ornamentation of segments as shown; EXP1 and EXP2 with outer distal process less developed than in P3, without pores, with inner distal frill, and with inner seta; EXP3 with subdistal outer pore, with three inner setae of which medial visibly stronger, two apical elements, and three outer spines. Endopod three-segmented, reaching tip of EXP2; spinular ornamentation of segments as shown; ENP1 shortest, ENP3 longest; ENP1 with small outer distal process, without pore, with inner pinnate seta; ENP2 with well-developed outer distal process, armature as in previous segment; ENP3 with distal processes as shown, with subdistal inner pore, with two inner setae, two apical elements, and one outer apical spine.

Setal formula of swimming legs as follows:

**Table:** 

	P1	P2	P3	P4
**EXP**	0,0,022	1,1,123	1,1,223	1,1,323
**ENP**	1,1,111	1,2,121	1,1,221	1,1,221

**P5** (Fig. 8C): Baseoendopod transversely elongate; with five setae, of which outermost shortest and set close to adjoining element, all setae whip-like without any transformation. Exopod oval, with some outer proximal spinules, with six setae, of which fourth from outer to inner margin shortest.

**P6** (Fig. 3B, C): Represented by a minute flap covering ventrolateral genital aperture, fused to somite, without surface ornamentation, with one slender seta.

***Male*.** Total body length measured from tip of rostrum to posterior margin of caudal rami ranging from 289 µm to 460 µm (mean, 377 µm; n, 7; total body length of allotype, 415 µm).

**Prosome**: As in female.

**Figure 9.**
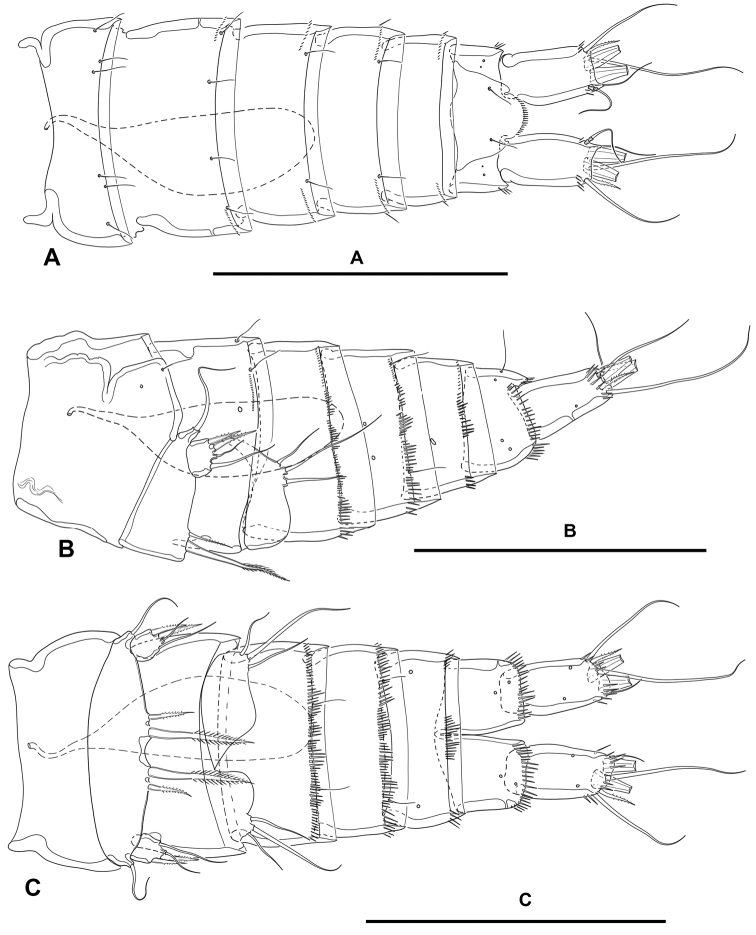
*Lonchoeidestenhelia prote* gen. et sp. nov., male **A** urosome, dorsal **B** urosome, lateral **C** urosome, ventral. Scale bars: 100 µm (**A–C**).

**Urosome** (Fig. 9A–C): Largely as in female except for genital somite and third urosomite separated, and for lateral and ventral spinular ornamentation.

**Caudal rami** (Fig. 9A–C): As in female.

**Sexual dimorphism**: Expressed in A1, P2ENP, P5, and P6.

**Figure 10.**
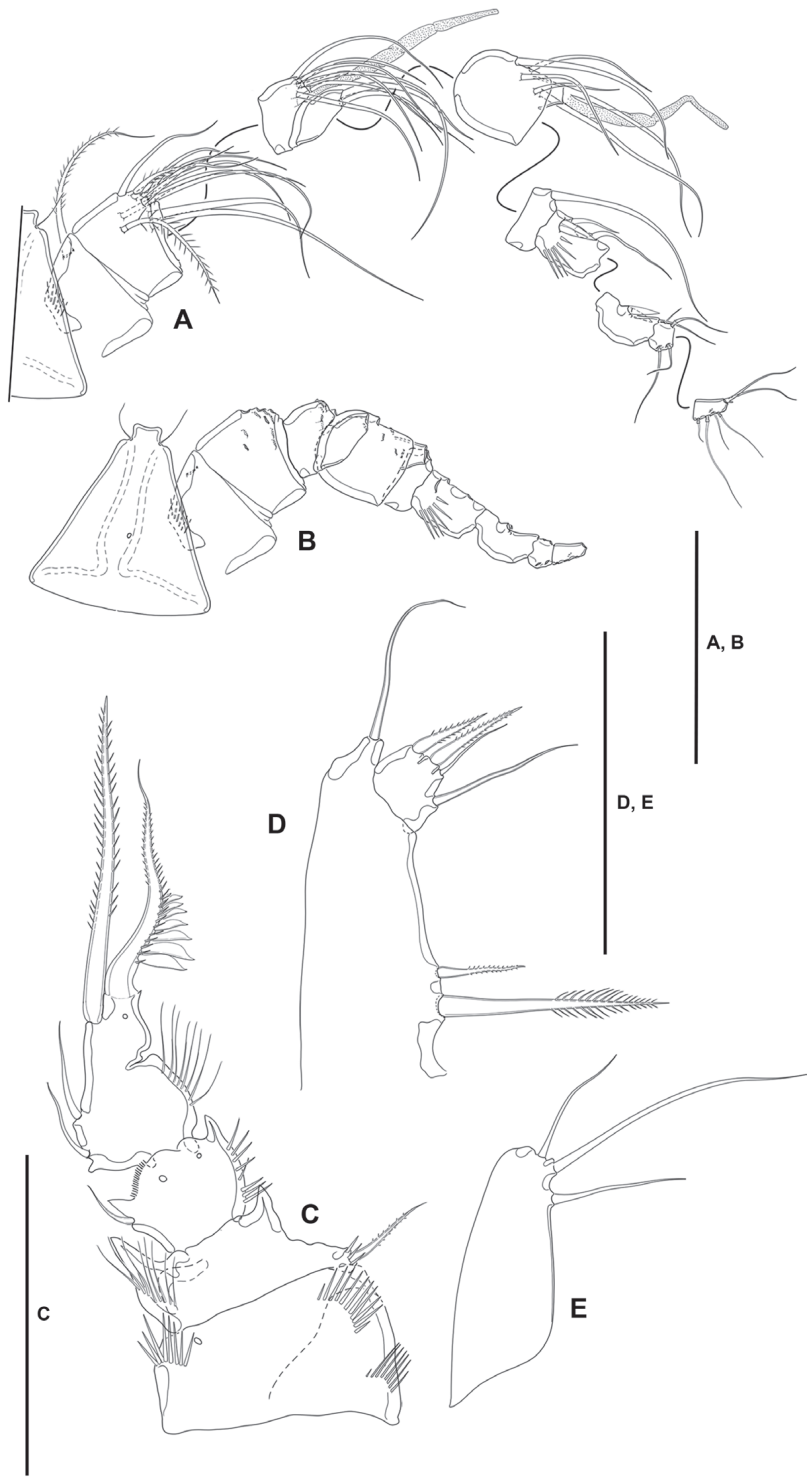
*Lonchoeidestenhelia prote* gen. et sp. nov., male **A** rostrum antennule with armature **B** rostrum and antennule without armature **C**P2ENP, anterior **D**P5, anterior **E**P6, anterior. Scale bars: 50 µm (**A–E**).

**Antennule** (Fig. 10A, B): 10-segmented, haplocer. All segments smooth except for first and seventh segment with spinules as shown. All setae smooth except for one and two pinnate setae on first and second segments, respectively, and one and two modified spine-like elements on seventh and eighth segments. Armature formula: 1(1); 2(11); 3(7+ae); 4(2), 5(5+(1+ae)); 6(1); 7(3); 8(3); 9(4);10(4+acro). Acrothek consisting of two setae and one minute aesthetasc fused at their bases.

**Antenna, mandible, maxillule, maxilla, and maxilliped**: As in female.

**P1**: As in female.

**P2**: EXP (not shown) as in female. ENP (Fig. 10C) sexually dimorphic; basis, coxa and ENP1 largely as in female; ENP2 transformed, proximal half globular, distal half triangular, with inner notch indicating former division between ENP2 and ENP3, proximal half with inner long setules, distal half with subdistal pore, proximal half with two inner (one proximal and one medial) small setae, distal half with one inner subdistal strong pinnate element, and one apical sigmoid pinnate process with proximal row of outer spinules modified into lanceolate ornaments.

**P3 and P4**: As in female.

**P5** (Figs 9B, C, 10D): Baseoendopods of both legs fused medially forming a continuous plate; each endopodal lobe with two setae, of which inner well-developed, outer small. Exopod small, discrete; with four elements, of which two outermost spine-like, third element from outer to inner margin shortest, innermost seta longest.

**P6** (Fig. 9B, C, 10E): Asymmetrical, only one leg functional; each leg with three setae, of which medial longest, inner and outer elements subequal in length.

**Figure 11.**
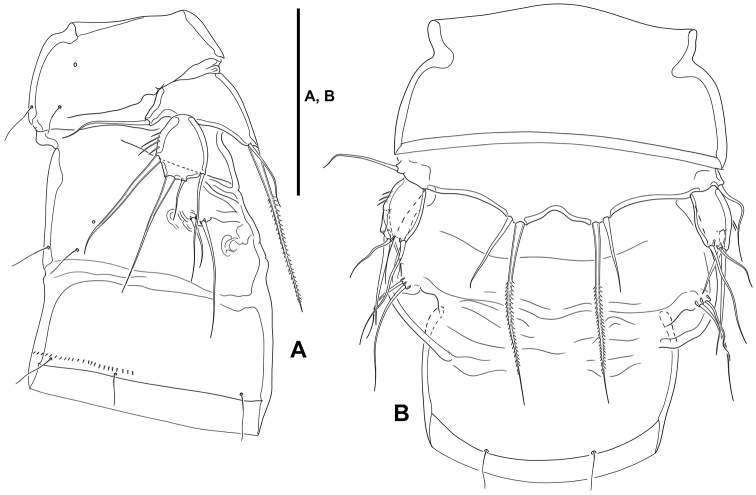
*Lonchoeidestenhelia prote* gen. et sp. nov., intersex individual **A**P5-bearing somite and double genital-somite, lateral **B**P5-bearing somite and double genital-somite, ventral. Scale bar: 50 µm (**A, B**).

**Variability.** One intersexual specimen (ICML–EMUCOP-180119-10) possesses female antennules, displays genital double-somite (Fig. 11B), and lacks dimorphism in swimming legs, but the P5 (Fig. 11A, B) seems more of the male type with exopod bearing four elements (but two outer elements long and seta-like), and both baseoendopods fused medially and with two setae (outer small, inner long and pinnate), and the P6 possesses two setae.

##### Etymology.

The specific epithet from the Greek *πρώτη*, prṓtē, first, makes reference to the first species of *Lonchoeidestenhelia* gen. nov. described so far. Gender feminine.

##### Type locality.

Mexico, Sinaloa State: Urías estuary, stn 2, 23.1587°N, 106.3326°W, depth 1.8 m, organic carbon content 3.99%, organic matter content 6.86%, sand 80.42%, clay 8.29%, silt 11.28%.

##### Other localities.

Mexico, Sinaloa State: Urías estuary, stn 1: 23.15194°N, 106.3128°W, depth 1.5 m, organic carbon content 3.74%, organic matter content 6.43%, sand 25.31%, clay 35.75%, silt 38.94%, stn 4: 23.1840°N, 106.3579°W, depth 0.7 m, organic carbon content 1.13%, organic matter content 1.94%, sand 82.44%, clay 8.27%, silt 9.29%, stn 7: 23.2174°N, 106.3917°W, depth 3.7 m, organic carbon content 5.59%, organic matter content 9.62%, sand 10.78%, clay 37.54%, silt 51.68%, stn 9: 23.1904°N, 106.4121°W, depth 5.4 m, organic carbon content 1.41%, organic matter content 2.43%, sand 64.81%, clay 8.09%, silt 27.11%, and stn 10: 23.1815°N, 106.4214°W, depth 6.0 m, organic carbon content 1.2%, organic matter content 2.07%, sand 69.12%, clay 7.91%, silt 22.97%.

#### Genus Willenstenhelia

Taxon classificationAnimaliaHarpacticoidaMiraciidae

Karanovic and Kim, 2014

6572B34A-6471-57D1-8615-A4EA3A8D12D9

##### Type species.

*Willenstenhelia thalia* Karanovic and Kim, 2014, by original designation.

##### Other species.

*Wi. minuta* (A. Scott, 1902) (= *D. minuta* A. Scott, 1902), *Wi. unisetosa* (Wells, 1967) (= Stenhelia (Delavalia) unisetosa Wells, 1967), *Wi. reducta* sp. nov., *Wi. urania* Karanovic and Kim, 2014, and *Wi. terpsichore* Karanovic and Kim, 2014.

#### Willenstenhelia reducta sp. nov.

Taxon classificationAnimaliaHarpacticoidaMiraciidae

6F5B5E5F-4D46-580A-843D-4DCC3D7BF7DE

http://zoobank.org/F5070C00-3D05-4EE1-9E9B-310C1E6335E8

Figs 12 , 13 , 14 , 15 , 16 , 17 , 18 , 19 , 20 , 21

##### Specimens examined.

One female holotype (ICML–EMUCOP-180119-25) from the type locality and one male allotype (ICML–EMUCOP-180119-26) from stn 4; four (ICML–EMUCOP-180119-31) and one (ICML–EMUCOP-180119-32) female paratypes from the type locality; one female and one male paratype (ICML–EMUCOP-180119-33), one female paratype (ICML–EMUCOP-180119-34), and two female paratypes (ICML–EMUCOP-180119-35) from stn 4; one female and one male paratype (ICML–EMUCOP-180119-36), one female paratype (ICML–EMUCOP-180119-37), and one CIV, one CV, one female and two male paratypes (ICML–EMUCOP-180119-38) from stn 9; one CIII, one CIV, and two female paratypes (ICML–EMUCOP-180119-39) from stn 10; all preserved in alcohol. Two dissected female paratypes (ICML–EMUCOP-180119-27, ICML–EMUCOP-180119-28) mounted onto eight and six slides, respectively, and one dissected male paratype (ICML–EMUCOP-180119-29) mounted onto three slides, all from stn 2; one dissected male paratype (ICML–EMUCOP-180119-30) from stn 4 mounted onto eight slides. 18 Jan. 2019. S. Gómez leg.

##### Differential diagnosis.

Stenheliinae: *Willenstenhelia*. Female antennule eight-segmented. Armature formula of P4EXP, 0,1,122. Female P5 baseoendopods not fused medially; endopodal lobe with two setae separated by wide gap, outer seta well-developed, inner seta minute; exopod with five setae, of which innermost as long as two outermost setae, middle seta smallest, inner neighboring seta longest. Male antennule ten-segmented. Male P5EXP discrete, with four elements, of which apical a strong spine, two medial ones small and subequal in length, innermost smallest arising midway inner margin; baseoendopods fused medially forming a continuous plate, each endopodal lobe with one strong spine-like element fused to endopod, both elements set close to each other.

##### Description.

**Figure 12.**
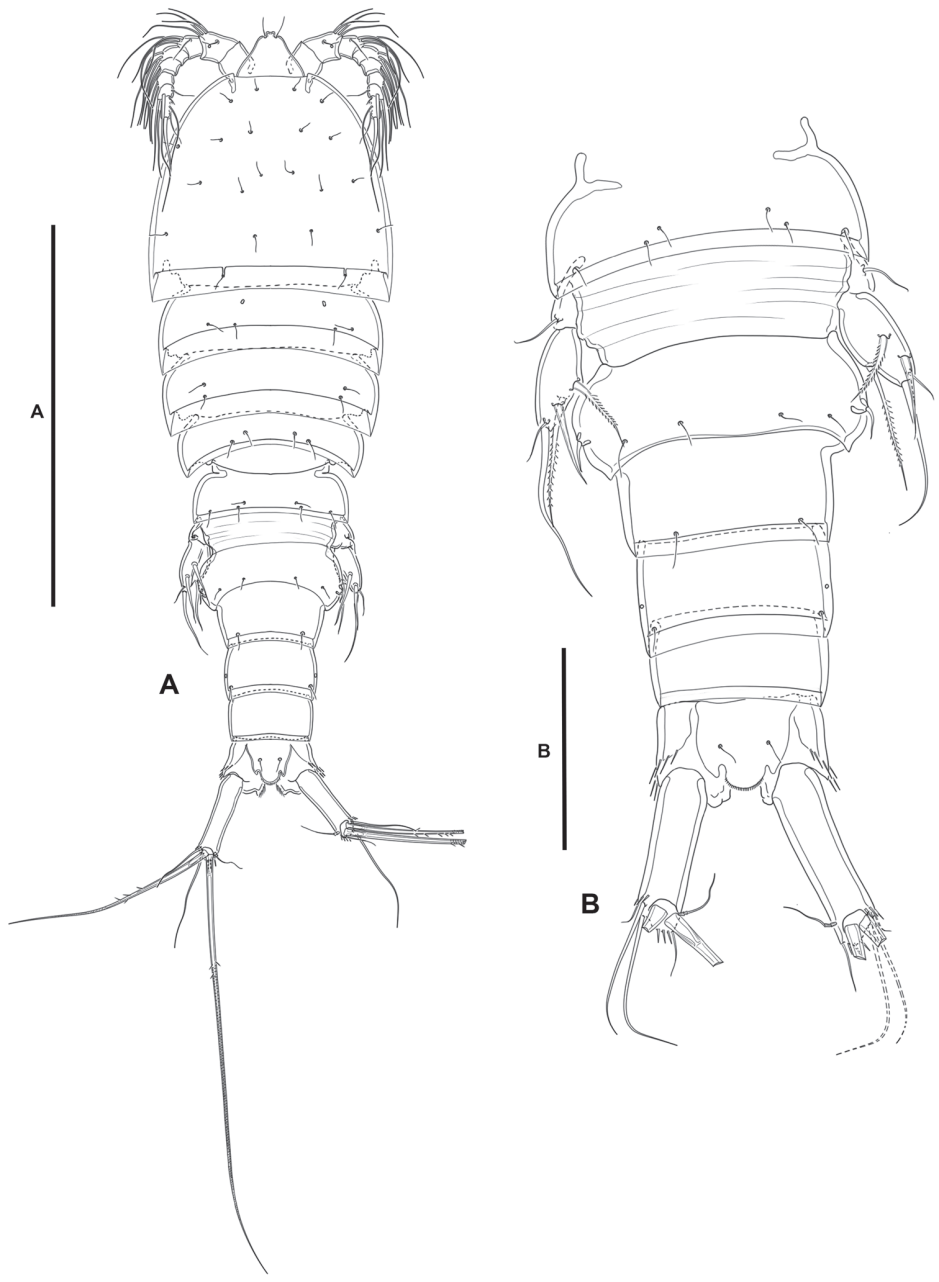
*Willenstenhelia reducta* sp. nov., female **A** habitus, dorsal **B** urosome, dorsal. Scale bars: 200 µm (**A**); 50 µm (**B**).

***Female*.** Total body length measured from tip of rostrum to posterior margin of caudal rami ranging from 445 µm to 510 µm (mean, 477 µm; n, 3; total body length of holotype, 510 µm); habitus (Fig. 12A) pyriform, widest at posterior end of cephalothorax in dorsal view, tapering posteriad.

**Prosome** (Fig. 12A): Consisting of cephalothorax with fused first pedigerous somite, and second to fourth free pedigerous somites, the latter without expansions nor spinular ornamentation; posterior hyaline frill of cephalothorax and pedigerous somites plain, of fourth pedigerous somite visibly narrower; integument smooth, weakly sclerotized.

**Figure 13.**
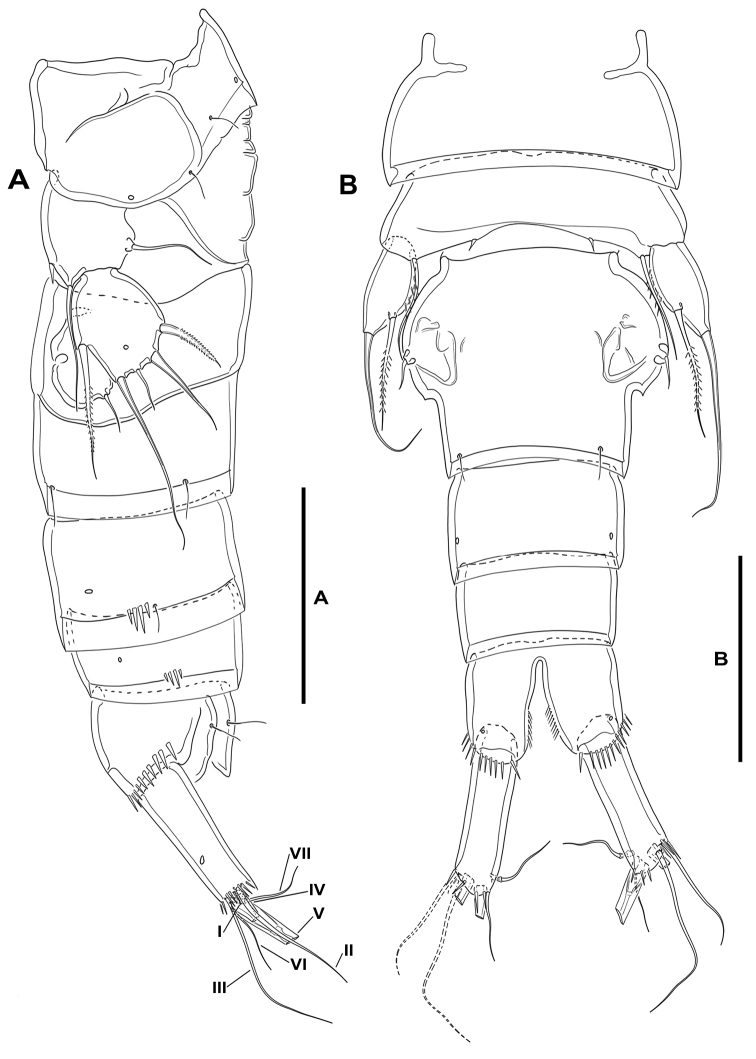
*Willenstenhelia reducta* sp. nov., female **A** urosome, lateral **B** urosome, ventral. Scale bars: 50 µm (**A, B**).

**Urosome** (Figs 12A, B, 13A, B): Consisting of fifth pedigerous somite (first urosomite), genital double-somite (genital second urosomite and third urosomite fused), two free urosomites, and anal somite; urosomites without expansions laterally nor dorsally; integument weakly sclerotized.

**First urosomite** (fifth pedigerous somite): Visibly narrower than preceding somites in dorsal view (Fig. 12A, B), without spinular ornamentation (Figs 12A, B, 13A, B).

**Genital double-somite**: Ca. 1.5× as wide as long, widest part at proximal half (Fig. 12A, B); separated dorsolaterally (Figs 12A, B, 13A), completely fused ventrally (Fig. 13B); without spinular ornamentation, with posterior sensilla as depicted.

**Fourth urosomite**: With short spinular row and pore laterally on each side (Fig. 13A) and with sensilla as shown.

**Fifth urosomite** (Fig. 13A): Without sensilla; spinular ornamentation and pores as in previous somite.

**Anal somite** (Figs 12A, B, 13A, B): Twice as long as broad, maximum breadth measured at proximal margin; maximum length measured at the middle from anterior margin of somite to distal margin of anal operculum, with row of dorsolateral spinules close to joint with caudal rami (Figs 12A, B, 13A), ventrally cleft medially (Fig. 13B), with one subdistal pore on each side, with spinular row close to joint with caudal rami; anal operculum (Fig. 12A, B) rounded, with minute spinules along its posterior margin, with one sensilla on each side.

**Caudal rami** (Figs 12A, B, 13B): Typically divergent, cylindrical, ca. 2.7× as long as broad, and 1.4× as long as anal somite in dorsal view; each ramus with one subdistal lateral pore (Fig. 13A); with spinules at base of setae I and II (Figs 12B, 13A), and ventrally (Fig. 13B); with seven elements; seta I very small, often masked behind spinules, ventral to seta II, the latter long, both arising subdistally on lateral margin; seta III ventral, subdistal, longer than seta II; seta IV and V situated distally, with fracture plane; seta VI issuing at inner distal corner; dorsal seta VII tri-articulate at base, situated subdistally close to inner margin.

**Rostrum** (Fig. 12A): Trapezoidal, not fused to cephalothorax, reaching tip of first antennular segment, bifid at tip, without pore, with two subdistal sensilla.

**Figure 14.**
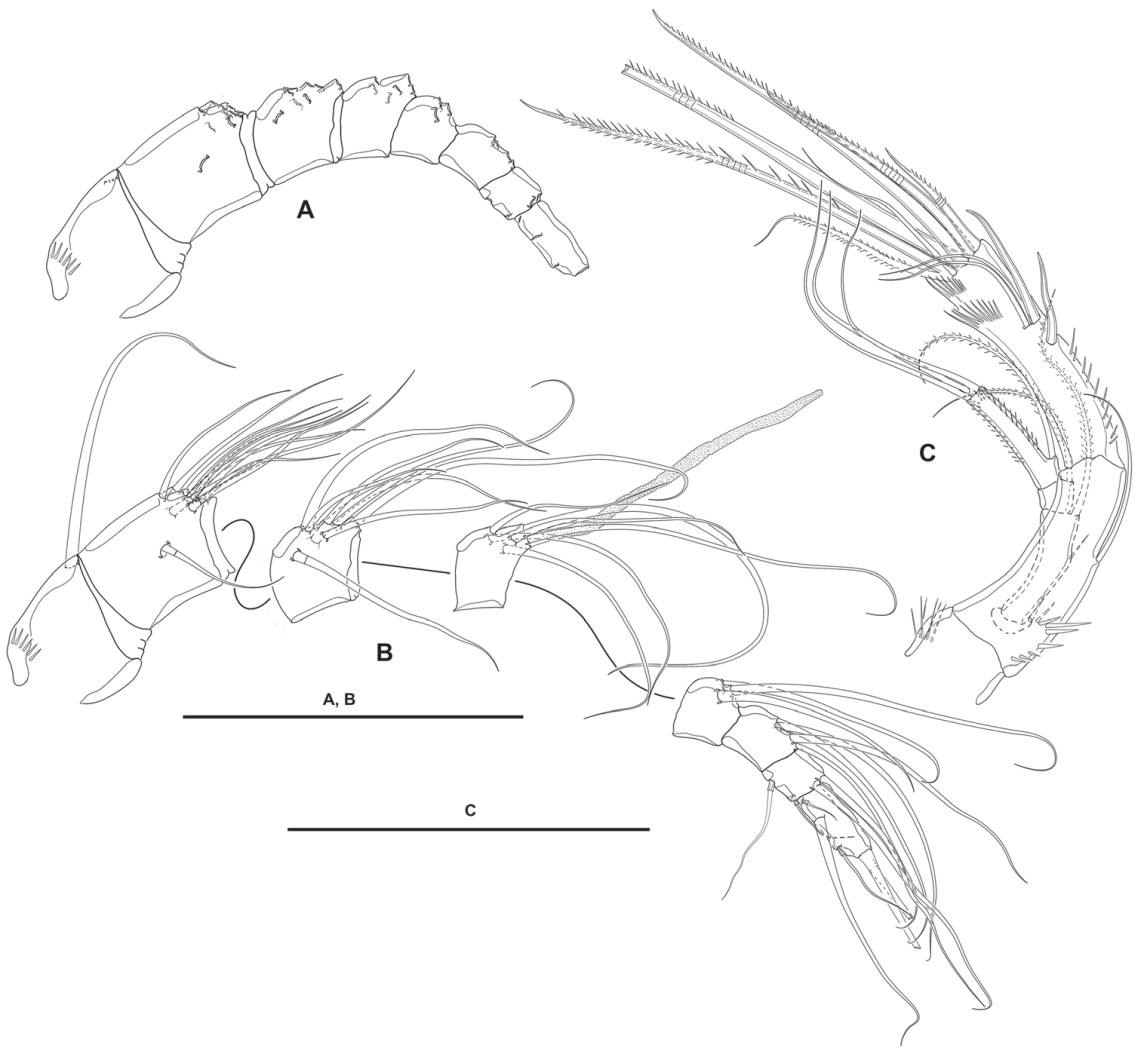
*Willenstenhelia reducta* sp. nov., female **A** antennule without armature **B** antennule with armature **C** antenna. Scale bars: 50 µm (**A–C**).

**Antennule** (Fig. 14A, B): Eight-segmented; all segments smooth, except for first segment with spinular row. All setae seemingly smooth; second and third segments each with one seta with fracture plane; seventh and eighth segments with two articulated setae each. Armature formula: 1(1); 2(11); 3(8); 4(5+(1+ae)), 5(3); 6(4); 7(4); 8(3+(2 setae fused basally)). Eighth segment seemingly without aesthetasc.

**Antenna** (Fig. 14C): Coxa small, with some outer spinules. Allobasis as long as free endopodal segment, inner margin with some proximal spinules, with one (pinnate?) abexopodal seta arising midway on inner margin. Exopod three-segmented, issuing proximally; first and third segments longest, each 3× as long as wide, second segment shortest and ca. 1.5× as long as broad; first and second segments with one pinnate seta each, spinular ornamentation of first segment a single spinule, second segment unornamented; third segment with small spinules as shown, with one lateral pinnate element proximally, and three distal setae, of which two fused basally. Free endopodal segment elongate, inner margin with proximal row of small spinules, with medial and subdistal inner fringe; armature consisting of two lateral spines and two accompanying setae, one geniculate inner distal element, three geniculate distal spines (of which innermost shortest) and one slender seta, and one outer distal geniculate seta fused basally to pinnate element.

**Figure 15.**
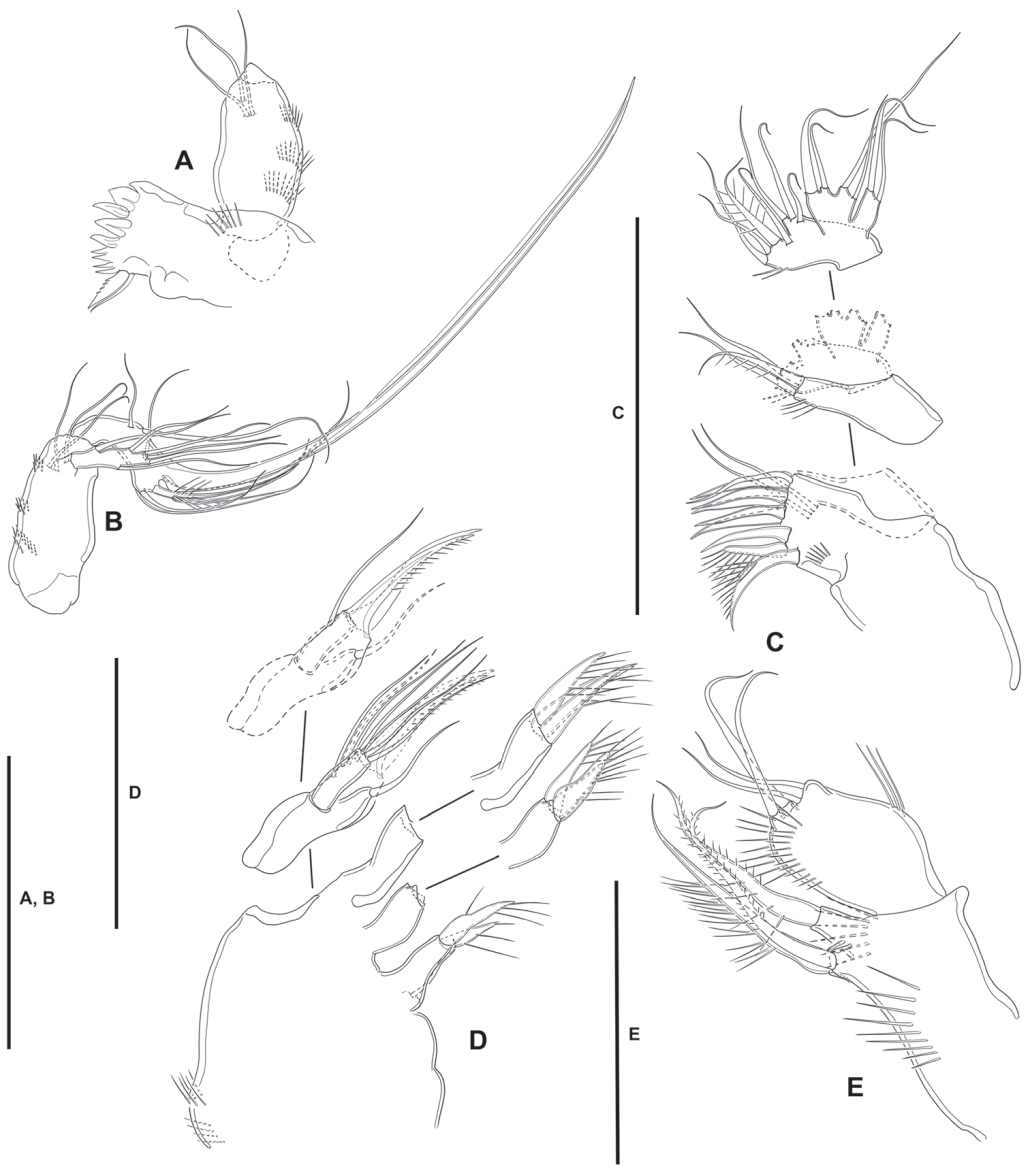
*Willenstenhelia reducta* sp. nov., female **A** mandibular coxa and basis **B** another mandibular basis, exopod and endopod **C** maxillule **D** maxilla **E** maxilliped. Scale bars: 50 µm (**A–C**); 25 µm (**D, E**).

**Mandible** (Fig. 15A, B): With relatively short coxa. Gnathobasis wide; with two strong bicuspidate teeth, several smaller bicuspidate teeth, some spinules, and one strong lanceolate element accompanied by slender seta. Basis elongate, tapering distally, with transverse spinular rows as shown, with three subdistal setae. Exopod arising from short pedestal, one-segmented, elongate, ca. 3.5× as long as wide, tapering distally; with three lateral and three apical slender setae, none of which fused basally. Endopod recurved, twisted over exopod; with three lateral setae, and five distal elements (one smooth and one long pinnate element, two long setae fused basally and fused to endopod, and one long element fused to endopod and with hyaline flange in middle part).

**Maxillule** (Fig. 15C): Arthrite of praecoxa with two surface setae and seven bare distal elements (one of which a slender seta arising next to ventralmost spine), one spinulose dorsal spine, and one lateral spinulose recurved seta. Coxal endite with three setae. Basis with two endites; proximal endite with four, distal endite with three slender setae. Exopod and endopod fused basally, and fused also to basis, each ramus one-segmented; endopod larger than exopod, with four setae; exopod small, with two setae.

**Maxilla** (Fig. 15D): Large syncoxa with outer spinules as shown; with three endites; proximal endite smallest, with one slender proximal and two strong distal setae; middle and distal endites elongate, the latter slightly longer, with three elements each as figured. Basis drawn out into strong claw, additionally with strong spine and two slender setae, one of which arising from elongate setophore. Endopod one-segmented, 2× as long as wide, with six slender setae.

**Maxilliped** (Fig. 15E): Non-prehensile. Syncoxa rectangular, ca. 2× as long as wide; with anterior and posterior spinules as shown; with two proximal elements arising from short pedestals, and one apical element arising from long pedestal. Basis shorter than syncoxa, oval; with inner and outer spinules as depicted, and two slender distal setae subequal in length. Endopod absorbed into basis, with two slender setae.

**Figure 16.**
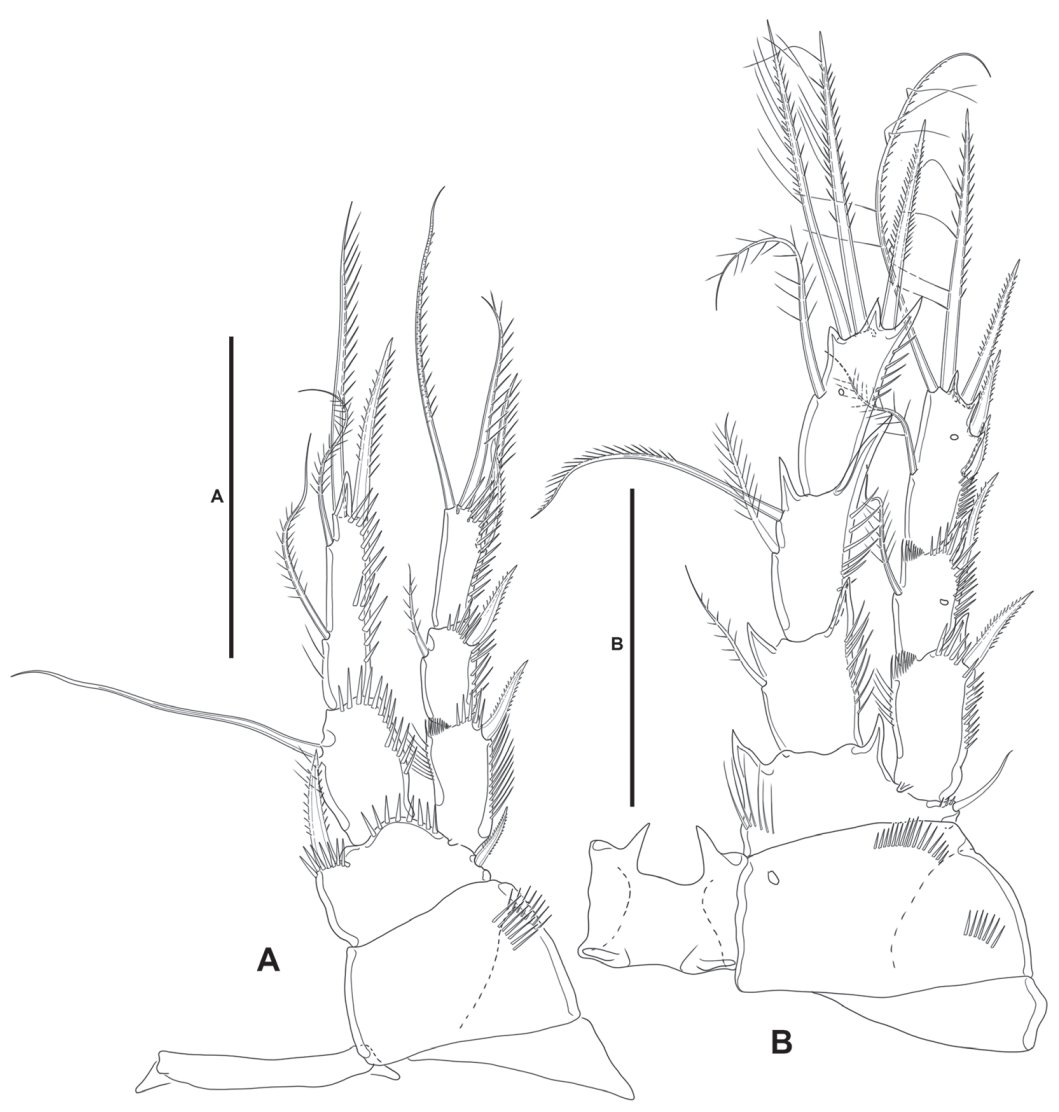
*Willenstenhelia reducta* sp. nov., female **A**P1, anterior **B**P2, anterior. Scale bars: 50 µm (**A, B**).

**P1** (Fig. 16A): Intercoxal sclerite narrow and elongate, without surface ornamentation. Praecoxa triangular, unornamented. Coxa quadrate, with spinular rows as shown. Basis with inner robust and pinnate spine, and outer smaller pinnate spine, with strong spinules at the base of inner spine and between rami. Exopod three-segmented, as long as endopod; first and third segments longest, second segment shortest; all segments without outer nor inner acute distal processes; with spinular ornamentation as shown; first segment without, second segment with inner seta, third segment with four elements. Endopod two-segmented; ENP1 reaching slightly beyond EXP1, ca. 1.5× as long as wide, and 0.7× as long as ENP2, with outer and distal spinules as depicted; with one inner long seta; ENP2 longer than ENP1, with outer and distal spinules as shown, with one inner proximal, one inner subdistal, and two apical elements of which outermost a spine.

**P2** (Fig. 16B): Intercoxal sclerite not transversely elongate, trapezoidal, with strong pointed process on distal outer corners, without surface ornamentation. Praecoxa triangular, unornamented. Coxa massive, quadrate, with outer spinules proximally and subdistally, with one pore close to inner distal margin. Basis with outer setiform element with small spinules at its base, and strong acute process between rami and on inner distal corner, the latter with slender spinules proximally. Exopod three-segmented, shorter than endopod, reaching middle of ENP3; spinular ornamentation of segments as shown; EXP1 and EXP2 with inner distal frill, with outer acute distal process, EXP1 without, EXP2 with medial pore and inner seta; EXP3 with processes as shown, with subdistal pore, with one inner and two apical setae, and three outer spines. Endopod three-segmented; ENP1 shortest, ENP2 and ENP3 subequal in length; spinular ornamentation of segments as shown; ENP1 and ENP2 with inner and outer acute processes, ENP1 with one, ENP2 with two inner seta; ENP3 with distal processes as shown, with medial pore, with one inner seta, two apical elements, and one outer distal spine.

**Figure 17.**
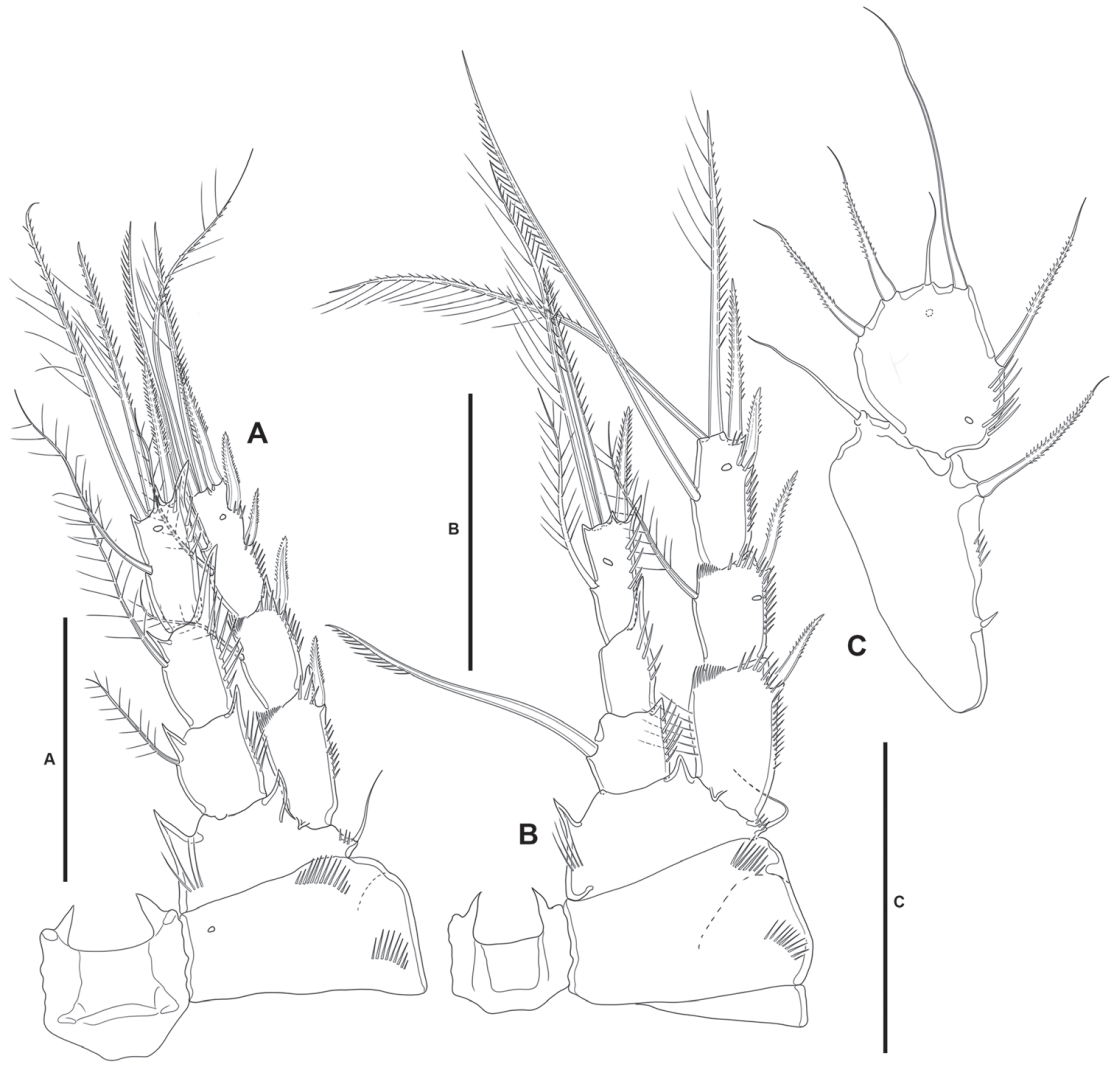
*Willenstenhelia reducta* sp. nov., female **A**P3, anterior **B**P4, anterior **C**P5, anterior. Scale bars: 50 µm (**A–C**).

**P3** (Fig. 17A): Intercoxal sclerite not transversely elongate, trapezoidal, with strong pointed process on distal outer corners, without surface ornamentation. Coxa as in P2. Basis as in P2, but with somewhat more slender outer seta. Exopod three-segmented, as long as ENP; spinular ornamentation of segments as shown; EXP1 and EXP2 with outer acute distal process, without pores, with inner distal frill, EXP1 without, EXP2 with inner seta; EXP3 with subdistal pore, with two inner setae of which the distal is visibly stronger, two apical elements, and three outer spines. Endopod three-segmented; ENP1 shortest, ENP3 longest; spinular ornamentation of segments as shown; ENP1 with small outer and inner distal processes, with inner seta; ENP2 with well-developed outer and small inner distal process, with inner seta; ENP3 with distal processes as shown, with subdistal pore, with one inner and two apical setae, and one outer apical spine.

**P4** (Fig. 17B): Intercoxal sclerite not transversely elongate, trapezoidal, with strong pointed process on distal outer corners; without surface ornamentation. Praecoxa triangular, unornamented; coxa and basis as in P3 except for comparatively smaller inner distal process of basis. Exopod three-segmented, longer than ENP; spinular ornamentation of segments as shown; EXP1 and EXP2 with outer distal process less developed than in P3, with inner distal frill, EXP1 without, EXP2 with subdistal pore and inner seta; EXP3 with subdistal pore, with one inner seta, two apical elements, and two outer spines. Endopod three-segmented, reaching proximal third of EXP3; ENP1 shortest, ENP3 longest; spinular ornamentation of segments as depicted; ENP1 with small outer distal process, with inner long stiff pinnate element; ENP2 with well-developed outer distal process, without inner armature; ENP3 with distal processes as shown, with subdistal pore, with one inner seta, two apical elements, and one outer apical spine.

Setal formula of swimming legs as follows:

**Table:** 

	**P1**	**P2**	**P3**	**P4**
**EXP**	0,1,022	0,1,123	0,1,223	0,1,122
**ENP**	1,211	1,2,121	1,1,121	1,0,121

**P5** (Fig. 17C): Baseoendopod transversely elongate; endopodal lobe with two setae, of which outer well-developed, inner minute, both separated by wide gap. Exopod trapezoidal, with some inner proximal spinules; with five setae, of which outermost, medial outer, and innermost setae pinnate and subequal in length, middle seta shortest and slender, medial inner seta bare and longest.

**P6** (Figs 12B, 13A, B): Represented by a minute flap covering ventrolateral genital aperture, fused to somite, without surface ornamentation, with one small seta.

***Male*.** Total body length measured from tip of rostrum to posterior margin of caudal rami ranging from 251 µm to 363 µm (mean, 309 µm; n, 5; total body length of allotype, 363 µm).

**Prosome**: As in female.

**Figure 18.**
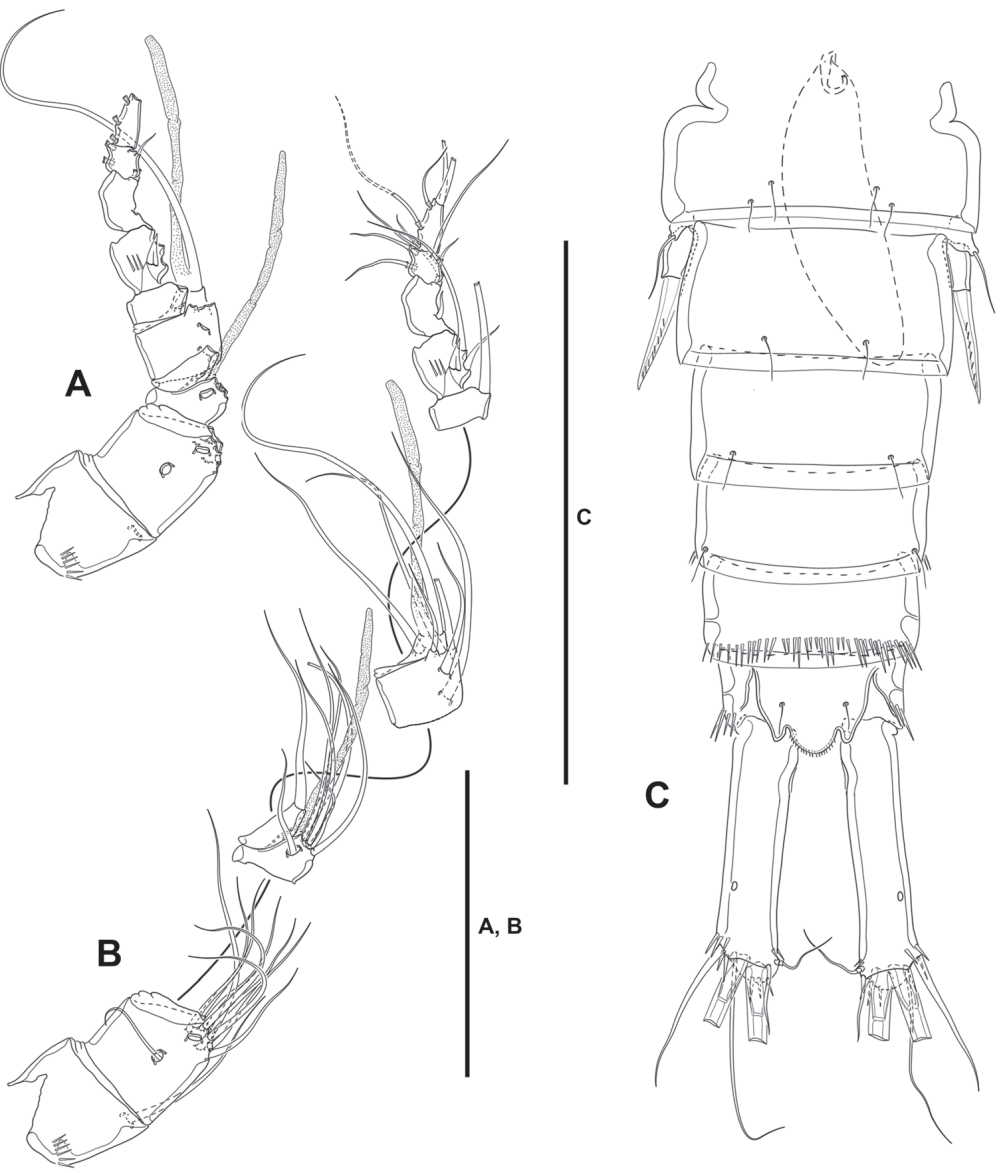
*Willenstenhelia reducta* sp. nov., male **A** antennule without armature **B** antennule with armature **C** urosome, dorsal. Scale bars: 50 µm (**A, B**); 100 µm (**C**).

**Figure 19.**
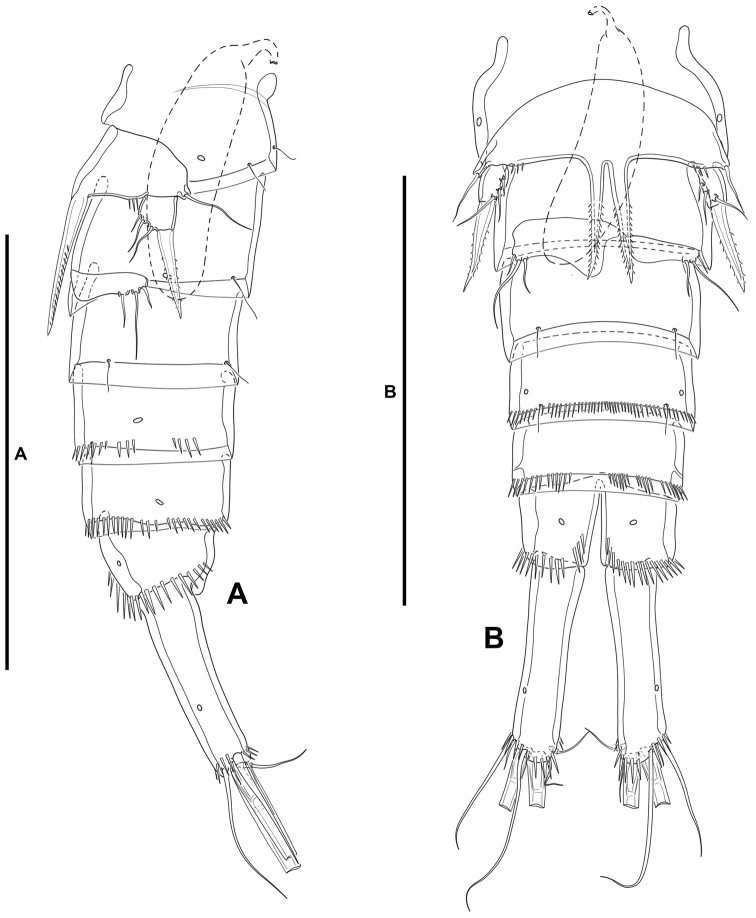
*Willenstenhelia reducta* sp. nov., male **A** urosome, lateral **B** urosome, ventral. Scale bars: 100 µm (**A, B**).

**Urosome**: Largely as in female except for genital and third urosomites separated, and spinular ornamentation of fourth and fifth urosomites (Figs 18C, 19A, B).

**Caudal rami** (Figs 18C, 19A, B): As in female.

**Sexual dimorphism**: Expressed in A1, P2ENP, P3, P4, P5, and P6.

**Antennule** (Fig. 18A, B): 10-segmented, haplocer. All segments smooth except for first and seventh segment with spinules as shown. All setae seemingly smooth; with one modified spine-like element on seventh segment. Armature formula: 1(1); 2(11); 3(7+ae); 4(1), 5(6+(1+ae)); 6(1); 7(3); 8(2); 9(4);10(3+2 setae fused basally).

**Antenna, mandible, maxillule, maxilla, and maxilliped**: As in female.

**P1**: As in female.

**Figure 20.**
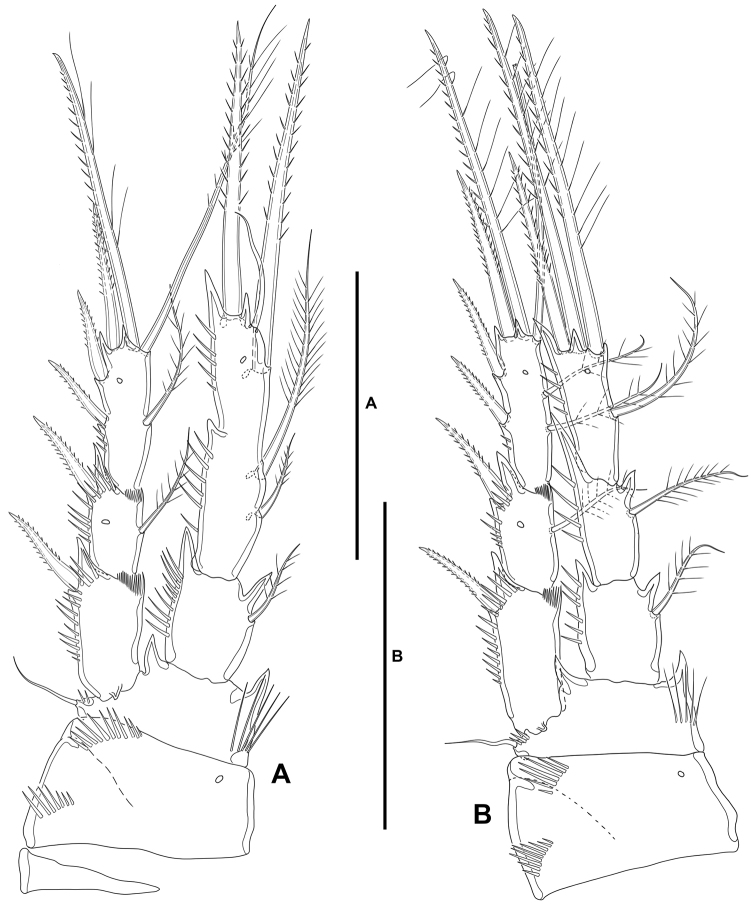
*Willenstenhelia reducta* sp. nov., male **A**P2, anterior **B**P3, anterior. Scale bars: 50 µm (**A, B**).

**P2** (Fig. 20A): Praecoxa, coxa, basis, and exopod as in female. Endopod sexually dimorphic, two segmented; first segment as in female; second segment with one proximal and one subdistal longitudinal row of spinules separated by medial process indicating division between ENP2 and ENP3 of the female ENP, with two inner elements homologues to the inner elements of the female ENP2, with subdistal pore, with one inner subdistal strong outer element, and one inner apical slender seta, and one apical outer spine-like element.

**P3** (Fig. 20B): Largely as in female, the general shape of the distalmost inner seta of EXP3 the only difference detected: very long, and pinnate element as in the female, but a visibly shorter and plumose seta in the male.

**Figure 21.**
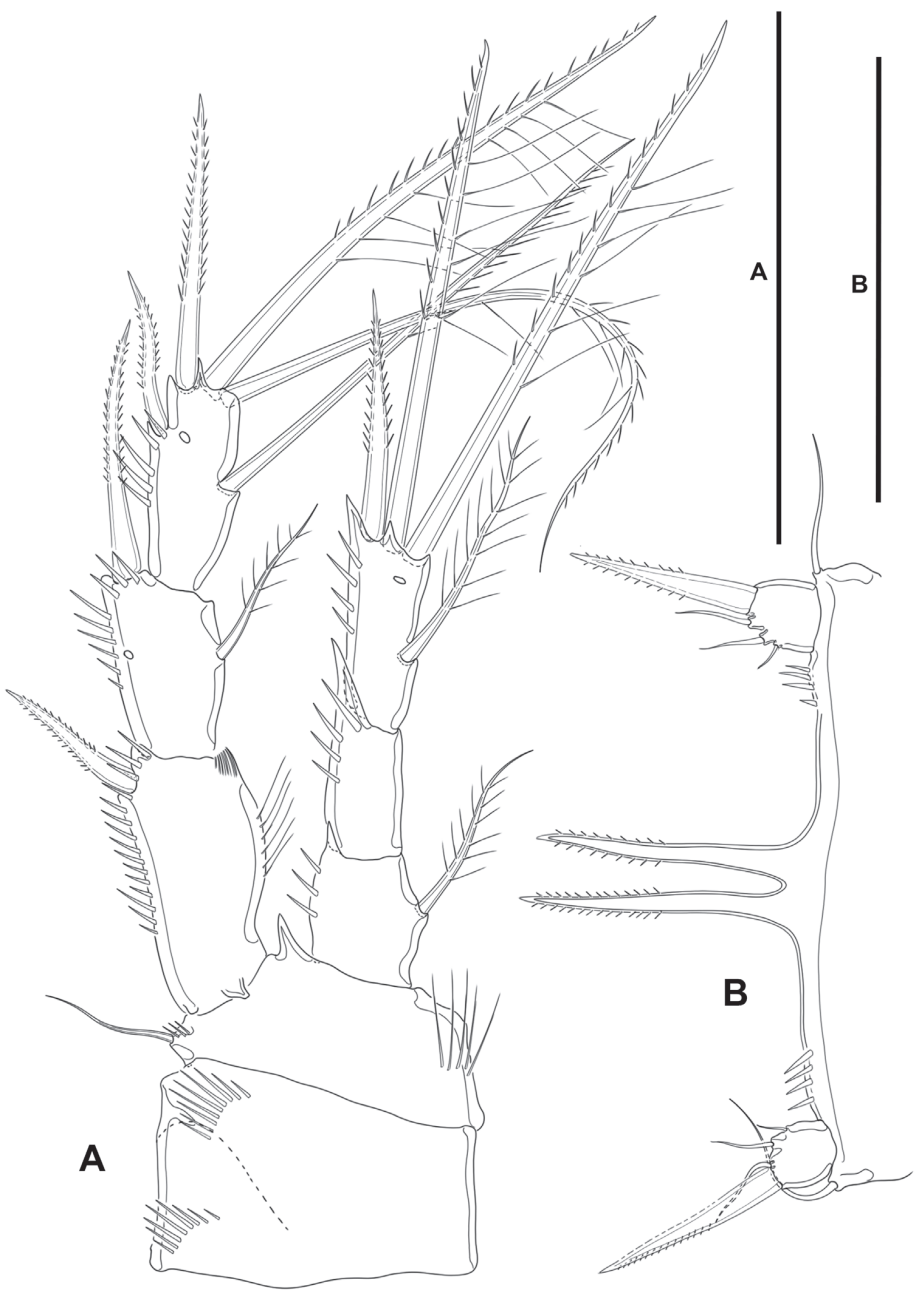
*Willenstenhelia reducta* sp. nov., male **A**P4, anterior **B**P5, anterior. Scale bars: 50 µm (**A, B**).

**P4** (Fig. 21A): Largely as in female, the relative length and shape of the inner seta on ENP1, inner seta of EXP2, and outer spine of EXP2 the only differences detected: inner seta of ENP1 very long and stiff in the female, but comparatively shorter, slender and plumose in the male; inner seta on the female EXP2 reaching beyond tip of EXP3 in the female, but visibly shorter in the male; outer spine of EXP2 of normal length not reaching tip of EXP3 in the female, but comparatively longer and reaching beyond tip of EXP3 in the male.

**P5** (Figs 19A, B, 21B): Baseoendopods of both P5 fused forming a continuous plate; each endopodal lobe with short row of spinules close to insertion of exopod, and with one element (the two elements are set very close together) fused to endopodal lobe. Exopod small, discrete; with four elements, of which apical a strong spine, two medial ones small and subequal in length, innermost smallest arising midway inner margin.

**P6** (Fig. 19A, B): Asymmetrical; each leg with three setae, of which medial longest, outer shortest.

##### Variability.

No variability was observed in the inspected material.

##### Etymology.

The specific epithet from the Latin *reducta*, reduced, in reference to the reduced inner seta on the female P5 baseoendopod, and to the reduced armature complement on the female and male P5BENP. It is an adjective in the nominative singular; gender feminine.

##### Type locality.

Mexico, Sinaloa State: Urías estuary, stn 2: 23.1587°N, 106.3326°W, depth 1.8 m, organic carbon content 3.99%, organic matter content 6.86%, sand 80.42%, clay 8.29%, silt 11.28%.

##### Other localities.

Mexico, Sinaloa State: Urías estuary, stn 4: 23.1840°N, 106.3579°W, depth 0.7 m, organic carbon content 1.13%, organic matter content 1.94%, sand 82.44%, clay 8.27%, silt 9.29%, stn 9: 23.1904°N, 106.4121°W, depth 5.4 m, organic carbon content 1.41%, organic matter content 2.43%, sand 64.81%, clay 8.09%, silt 27.11%, stn 10: 23.1815°N, 106.4214°W, depth 6.0 m, organic carbon content 1.20%, organic matter content 2.07%, sand 69.12%, clay 7.91%, silt 22.97%.

## Discussion

### Affinities of *Lonchoeidestenhelia prote* sp. nov.

In their paper, Mu and Huys (2002) proposed the abandonment of the traditional subgeneric division of the genus *Stenhelia* and restricted the genus to a core of species of the former subgenus Stenhelia (Stenhelia) Boeck, 1865, the type species *S. gibba* Boeck, 1865, and *S. proxima* Sars, 1906, *S. curviseta* Lang, 1936, *S. divergens* Nicholls, 1940, *S. peniculata* Lang, 1965, *S. pubescens* Chislenko, 1978, *S. sheni* Mu and Huys, 2002, and *S. taiae* Mu and Huys, 2002 (Mu and Huys 2002, Karanovic et al. 2014). Additionally, Mu and Huys (2002) reinstated the genus *Beatricella* for S. (S.) aemula T. Scott, 1893, created the genus *Anisostenhelia* for *S. asetosa* Thistle and Coull, 1979, and reassigned *S. diegensis* Thistle and Coull, 1979 *Delavalia* Brady, 1868. In their paper, Mu and Huys (2002) proposed the monophyly of *Stenhelia* based on the presence of a modified seta on the P5 baseoendopodal lobe (second innermost seta in the female; innermost element in the male) and on the probable presence of a hyaline flange on the P2ENP2 in the male of all its species. Their view was later corroborated by Karanovic et al. (2014). Additionally, Mu and Huys (2002) suggested a sister-group relationship with *Anisostenhelia* by the synapomorphic (i) loss of one inner seta on the P2 EXP3, resulting in the armature formula 123, (ii) loss of one inner seta on P3 EXP3, resulting in the armature formula 223, (iii) P2–P3ENP3 produced into an apical spinous process, resulting in the displacement of the outer spine to an apical situation, and both apical setae to the inner (subapical) margin, (iv) male P5EXP with two outermost elements modified into spines, (v) innermost element of male P6 modified into an outwardly recurved spine, and (vi) anal operculum absent. Also, Mu and Huys (2002) detected a potential synapomorphy for *Stenhelia*, *Anisostenhelia*, and *Beatricella*, the loss of the inner seta on the P1 EXP2. The genus *Anisostenhelia* was defined by Mu and Huys (2002) by the apomorphic (i) loss of the inner seta on P2–P4 EXP1, (ii) basal part of both apical setae on the female P2ENP3 typically swollen, (iii) male sexual dimorphism expressed in the modification of the outer spine of the P4ENP into a strongly recurved spine, and (iv) male P5 with exopod and baseoendopod fused. Finally, Mu and Huys (2002) defined the genus *Beatricella* by the apomorphic (i) male P2ENP drawn out into sigmoid finely pinnate process with long outer spinules, (ii) P4ENP1 with very long stout seta, and (iii) male P5EXP and baseoendopod fused and outermost exopodal element modified into strong spine.

*Lonchoeidestenhelia* gen. nov. shares the potentially synapomorphic loss of the inner seta on the P1 EXP2 (Mu and Huys 2002) with *Stenhelia*, *Anisostenhelia*, and *Beatricella*. *Lonchoeidestenhelia* gen. nov. seems to be more closely allied to *Stenhelia–Anisostenhelia* than to *Beatricella*. *Stenhelia*, *Anisostenhelia*, and *Lonchoeidestenhelia* gen. nov. share (i) the apomorphic loss of one inner seta on the P2 EXP3 (formula 123) and P3 EXP3 (formula 223), *Beatricella* displays a more primitive condition, with armature complement of 223 and 323 on the P2 EXP3 and P3 EXP3, respectively, (ii) P2ENP3 with apical outer spinous process with subsequent displacement of the outer spine to an apical situation, and medial and inner distal setae to a subapical inner position, and (iii) male P5EXP with two outermost elements modified into spines; only outermost element modified into a spine in the male P5EXP of *Beatricella*. *Lonchoeidestenhelia* gen. nov. seems to be more closely related to *Anisostenhelia* than to *Stenhelia*, by the overall shape of the male P2ENP2, with proximal half globular, distal half triangular in these two species, but proximal half visibly less globular and gradually tapering distally in *Stenhelia* and *Beatricella*. *Lonchoeidestenhelia* gen. nov. share the plesiomorphic non-modified setae of the female P5 baseoendopod and lack of hyaline flange on the male P2ENP2 with *Anisostenhelia*, and *Beatricella*; the plesiomorphic presence of one inner seta on P2–P4 EXP1, the apical setae on the female P2ENP3 not swollen basally, and the outer spine of the male P4ENP3 not sexually dimorphic with *Stenhelia* and *Beatricella*; and the plesiomorphic normal (unmodified) setae of the male P6 with normal, and presence of anal operculum with *Beatricella*. Finally, *Lonchoeidestenhelia* gen. nov. is defined here by the autapomorphic modified (lanceolate) proximal spinules on the outer distal process of the male P2ENP2.

**Affinities of *Willenstenhelia reducta* sp. nov.** The genus *Delavalia* is not only the most species-rich genus within the Stenheliinae, but also the most morphologically diverse. Although past decades have witnessed important advancements in the study of the genus, its monophyletic status is far from resolved (Mu and Huys 2002, Willen 2002, Karanovic et al. 2014). Probably, the most important contribution towards the monophyly of the genera *Stenhelia* Boeck and *Delavalia* was that of Mu and Huys (2002) who, amongst other things, challenged and abandoned the subgeneric classification of the genus *Stenhelia*, and consequently gave the subgenus Stenhelia (Delavalia) full generic rank. The genus *Delavalia* remained, nevertheless, polyphyletic (Mu and Huys 2002, Karanovic et al. 2014). Later, Willen (2003) proposed six groups/subgroups upon (i) the shape of the anal operculum, (ii) the combination of a specialized setation pattern on the female P5, (iii) presence/reduction/absence of the distal inner setae on P2–P4 EXP3, (iv) shape of the male and female P5, and (v) reduction of the setation of swimming legs; Dahms and Bresciani (1993) and Dahms et al. (2005) proposed some apomorphies for the genus based on naupliar morphology. Some years later, Huys and Mu (2008) discussed Willen (2003) and Dahms et al. (2005), and presented a subdivision of the genus *Delavalia* based on (i) the segmentation pattern of the antennary exopod, and (ii) number of outer spines on P2–P4 EXP3. More recently, Karanovic and Kim (2014) proved the polyphyly of the genus and proposed three genera with two-segmented P1 endopods, *Wellstenhelia* Karanovic and Kim, 2014 for its type species *We. calliope* Karanovic and Kim, 2014, and *We. clio* Karanovic and Kim, 201, *We. erato* Karanovic and Kim, 2014, *We. euterpe* Karanovic and Kim, 2014, *We. melpomene* Karanovic and Kim, 2014, *We. qingdaoensis* (Ma and Li, 2011), *We. hanstroemi* (Lang, 1948), and *We. bocqueti* (Soyer, 1972), *Willenstenhelia* for its type species *Wi. thalia*, and *Wi. minuta*, *Wi. unisetosa*, *Wi. urania*, and *Wi. terpsichore*, and *Itostenhelia* Karanovic and Kim, 2014 for its type specie *I. polyhymnia* Karanovic and Kim, 2014, and *I. golikovi* (Chislenko, 1978) (= Stenhelia (Delavalia) golikovi Chislenko, 1978).

Karanovic and Kim (2014) gave a list of autapomorphies for *Willenstenhelia*. These are:

1. nner apical seta on the male P2 ENP2 shorter than outer spine,
2. outer spine on the male P4 EXP2 more sclerotized than other spines and strongly curved inwards,
3. female P5 BENP with three elements only, and with a large gap between the innermost one and the other two setae,
4. P4 ENP2 without inner armature, and
5. female P5 EXP with five setae only, of which innermost element displaced to the inner margin.

Additionally, they (Karanovic and Kim 2014) suggested that *D. palustris palustris* Brady, 1868 known from salt marshes of Northumberland and Durham, the widely distributed *D. palustris bispinosa* (Bodin, 1970) which could be a separate species or a species complex (Karanovic and Kim 2014), *D. clavus* (Wells and Rao, 1987), *D. paraclavus* (Wells and Rao, 1987), and *D. valens* (Wells and Rao, 1987) all known from Andaman and Nicobar Islands, might be distant relatives of *Willenstenhelia*. Interestingly, the above species, along with *D. schminkei* (Willen, 2002) and an unidentified species, “*St.* spec. 5”, both found in sediment samples from Papua New Guinea (Willen 2002, 2003), belong to Willen’s (2003) “*S. clavus* group”, which seems to be restricted the Indo-Pacific region (Willen 2002, 2003). The “*S. clavus* group” was defined by Willen (2002, 2003) upon:

1. the lack of inner armature on P2–P4 EXP1,
2. presence of one inner seta at most on P2 EXP3,
3. presence of two inner setae at most on P3 EXP3,
4. P3 ENP3 with one inner seta at most,
5. P4 EXP3 with two outer spines only,
6. loss of distalmost inner seta of P4 EXP3,
7. loss of inner armature of P4 ENP2,
8. presence of one inner seta at most on P4 ENP3,
9. baseoendopods of pair of female P5 fused medially,
10. outermost element on the male P5 EXP modified into a strong spine fused to segment (the male of D. valens remains unknown though), and
11. the non-prehensile maxilliped with globular allobasis (endopod absorbed into basis) (Willen 2002, 2003).

Similarly, Huys and Mu (2008) attributed *D. palustris palustris*, *D. incerta* (Por, 1964) known from the Israeli coasts, and *D. schminkei*, *D. clavus*, *D. paraclavus*, *D. valens*, and *D. unisetosa* (Wells, 1967) from Inhaca Island to their group III of the genus *Delavalia*, defined by the presence of 3, 3, 2 outer spines on P2–P4 EXP3. Karanovic and Kim (2014) reallocated *D. unisetosa* into *Willenstenhelia*.

Karanovic and Kim (2014) noted, that the assumed autapomorphic characters (iv) P4ENP2 without inner armature and (v) female P5EXP with five setae only, of which the innermost element is displaced to the inner margin, for *Willenstenhelia* might have evolved convergently in some other stenheliins. As an example of this, they mentioned the lack of inner armature on the P4ENP2 of *Muohuysia xylophila* (Hicks, 1988), a genus pertaining to a different lineage as evidenced by the three-segmented P1 endopods. The lack of inner armature on P4ENP2 is present, however, in some other species somehow related to *Willenstenhelia*, e.g., *D. schminkei*, *D. valens*, *D. clavus*, and *D. paraclavus* (Karanovic and Kim 2014), rendering the autapomorphic status of this character, doubtful, but apomorphic for *Willenstenhelia* if convergence is assumed. Similarly, the autapomorphic status of the reduction of the inner apical seta on the male P2ENP2, shorter than the apical outer spine, is questionable, since a similar condition is present in *D. oblonga* (Lang, 1965) (see Lang 1965: 248, fig. 137f), and is regarded here as apomorphic for *Willenstenhelia* if convergence is invoked.

To the best of my knowledge, the innermost seta of the pentasetose female P5EXP displaced to the inner margin of the ramus is present only in *Willenstenhelia*, and its autapomorphic status for the genus in confirmed. Also, I could not find any other species, for which the male is known, with the outer spine of the male P4 EXP2 comparatively more sclerotized and longer than in the female, and curved inwards, and its autapomorphic status for *Willenstenhelia* is provisionally accepted. Some other species in other related genera, e.g., *Wellstenhelia euterpe*, display a reduced armature complement of the female P5BENP from five to three setae, but *Willenstenhelia* is unique in the wide gap between the innermost element and its outer neighboring seta. This wide gap between the innermost element and its next outer neighboring seta is accepted here as autapomorphic for *Willenstenhelia*.

As with other stenheliins with a two-segmented P1 endopod, *Wi. reducta* sp. nov. was initially attributed to *Delavalia*. However, it was subsequently allocated into *Willenstenhelia* on account of (i) the pentasetose female P5EXP in which the innermost seta is displaced to the inner margin, (ii) the reduced armature complement of the female P5BENP, with two setae only, and with a wide gap between the innermost element and its outer neighboring seta, and (iii) presence of a sclerotized long and recurved outer spine on the male P4 EXP2.

The interspecific relationships amongst the species of *Willenstenhelia* are not clear. The Mexican *Wi. reducta* sp. nov. seems to belong to a core of species composed of *Wi. minuta*, *Wi. urania*, and *Wi. terpsichore* characterized by the strongly reduced inner seta of the female P5 baseoendopod. However, *Wi. reducta* sp. nov. is different from the other three species in the presence of one outer seta only on the discrete, not fused medially, baseoendopods of the female P5, i.e. *Wi. reducta* sp. nov. underwent loss of the outermost shorter seta of the female P5 baseoendopod which is still present in *Wi. minuta*, *Wi. urania*, and *Wi. terpsichore*, and unlike these three species, both baseoendopods of the female P5 of *Wi. reducta* sp. nov. are not fused medially. The loss of the outermost seta of the female P5 baseoendopod of *Wi. reducta* sp. nov. is regarded here as autapomorphic for the species. The innermost seta of the female P5EXP and the outermost seta of the female P5 baseoendopod of *Willenstenhelia thalia* underwent reduction, but the innermost seta of the female P5 baseoendopod is visibly larger than in *Wi. minuta*, *Wi. urania*, and *Wi. terpsichore*. The reduction of the innermost seta of the female P5EXP of *Wi. thalia* is considered here as autapomorphic for that species. *Willenstenhelia unisetosa* stands out for its three well-developed setae on the female P5 baseoendopod and for the discrete baseoendopods of the female P5 which are regarded here as the most plesiomorphic conditions. *Willenstenhelia unisetosa* and *Wi. reducta* sp. nov. share the discrete baseoendopods of the female P5. The male is known only for *Wi. thalia*, *Wi. terpsichore*, *Wi. unisetosa* and *Wi. reducta* sp. nov. *Willenstenhelia reducta* sp. nov. shares the male P5EXP with three small inner setae and the discrete apical spine with *Wi. unisetosa*, but it also shares the endopodal spines fused to the baseoendopod with *Wi. terpsichore*. The most plesiomorphic condition seems to be that of *Wi. thalia* with two (or three?) inner setae on the male P5 baseoendopod, and the inner medial seta of the exopod well-developed.

## Supplementary Material

XML Treatment for Lonchoeidestenhelia

XML Treatment for Lonchoeidestenhelia prote

XML Treatment for Willenstenhelia

XML Treatment for Willenstenhelia reducta
